# Multiplicity of Mathematical Modeling Strategies to Search for Molecular and Cellular Insights into Bacteria Lung Infection

**DOI:** 10.3389/fphys.2017.00645

**Published:** 2017-08-30

**Authors:** Martina Cantone, Guido Santos, Pia Wentker, Xin Lai, Julio Vera

**Affiliations:** Laboratory of Systems Tumor Immunology, Department of Dermatology, Friedrich-Alexander University Erlangen-Nürnberg and Universitätsklinikum Erlangen Erlangen, Germany

**Keywords:** systems biology, systems medicine, lung infection, mathematical modeling, Boolean network, ODE models, stochastic modeling, agent-based modeling

## Abstract

Even today two bacterial lung infections, namely pneumonia and tuberculosis, are among the 10 most frequent causes of death worldwide. These infections still lack effective treatments in many developing countries and in immunocompromised populations like infants, elderly people and transplanted patients. The interaction between bacteria and the host is a complex system of interlinked intercellular and the intracellular processes, enriched in regulatory structures like positive and negative feedback loops. Severe pathological condition can emerge when the immune system of the host fails to neutralize the infection. This failure can result in systemic spreading of pathogens or overwhelming immune response followed by a systemic inflammatory response. Mathematical modeling is a promising tool to dissect the complexity underlying pathogenesis of bacterial lung infection at the molecular, cellular and tissue levels, and also at the interfaces among levels. In this article, we introduce mathematical and computational modeling frameworks that can be used for investigating molecular and cellular mechanisms underlying bacterial lung infection. Then, we compile and discuss published results on the modeling of regulatory pathways and cell populations relevant for lung infection and inflammation. Finally, we discuss how to make use of this multiplicity of modeling approaches to open new avenues in the search of the molecular and cellular mechanisms underlying bacterial infection in the lung.

## Introduction

In a time of moon shooting projects to cure cancer (Nature Editorial, 2016), the reader may wonder why it remains interesting to deploy a “systemic approach” to deepen our understanding of bacterial lung infections. First, even nowadays two of the 10 most frequent causes of death worldwide are bacterial infections targeting the lungs, namely pneumonia and tuberculosis (WHO, 2017b). A few generations ago, respiratory infections used to claim the life of a significant fraction of infants, a problem circumvented in western countries with the emergence of antibiotics, sulfonamides and high quality health care, but still a dramatic reality in many developing countries. Second, elderly individuals and immunocompromised individuals face the challenge of repeated respiratory infections (Stupka et al., 2009). A similar problem is faced by immunocompromised populations (Conces, 1998).

Finally, bacteria resistant to antibiotics create new risks and motivate the struggle to create new antibiotics (Silver, 2011; WHO, 2017a).

**Figure 1:**
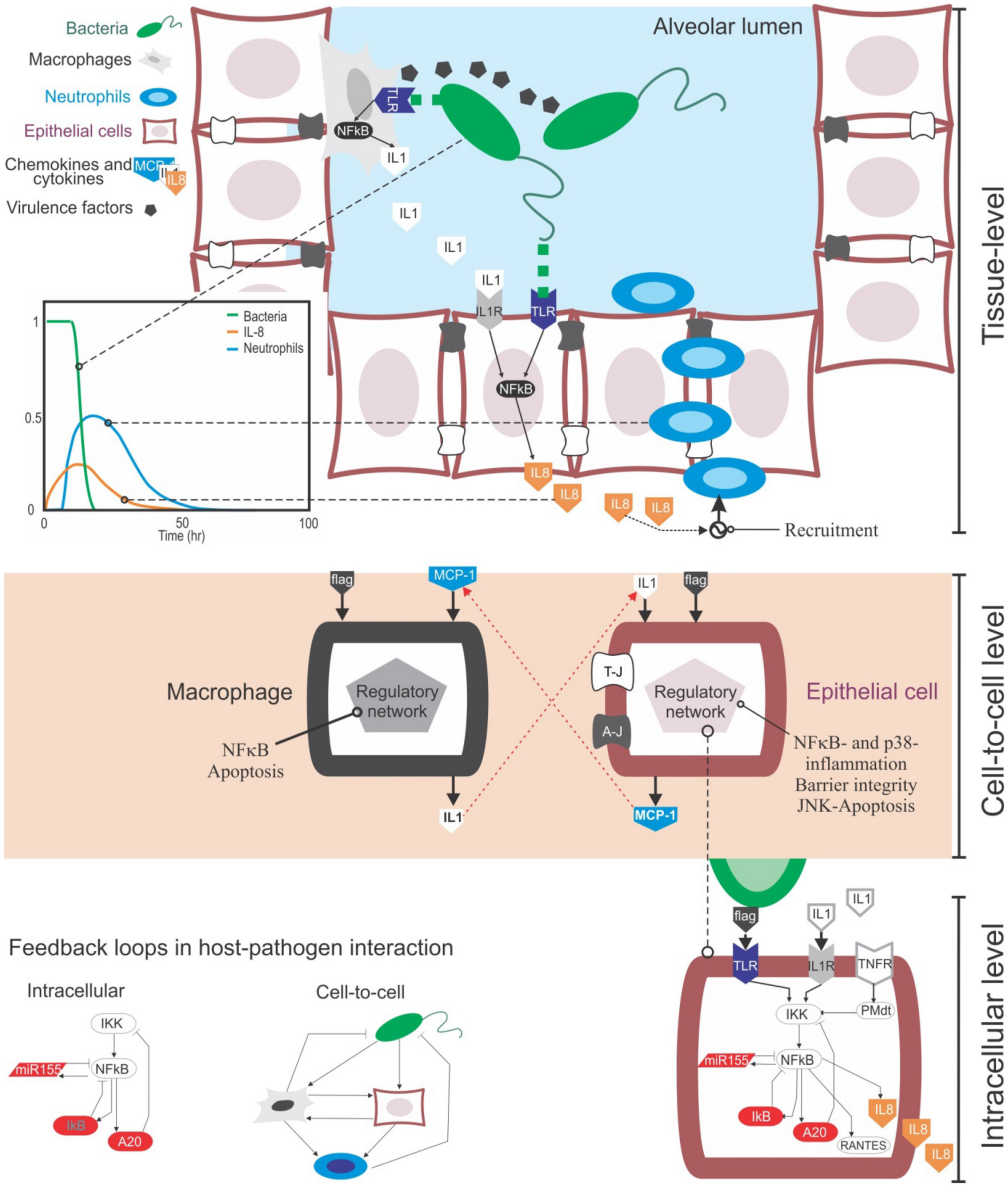
The multi-level complexity underlying the host-pathogen interaction in bacterial lung infection. **Top:** At the tissue level the infection involves the movement in the tissue compartment of multiple cell types, including bacteria, epithelial cells and immune cells like macrophages and neutrophils. During their movement, these cells interact with each other via physical contact (e.g., bacteria recognized by macrophages via TLR receptors) or through gradients of chemical signal secreted into the extracellular medium (chemokines from immune and epithelial cells, or virulence factors from bacteria). These events happen sequentially: for example, upon bacteria detection, epithelial cells secrete chemokines like IL-8 and CXCL5, and they guide neutrophils to the site of infection that can remove clear pathogens (see the plot). **Centre:** Cell-to-cell communications rely both on physical contact and the secretion of chemokines. Chemokines trigger the activation of distinctive, complex regulatory intracellular networks that can alter cell phenotypes or promote the secretion of more cytokines. For example, upon bacteria-mediated activation epithelial cells can secrete MCP-1, a chemokine that attracts macrophages. In turn, activated macrophages can secrete IL-1β, which activates epithelial cells. **Bottom:** At the intracellular level, the activation of epithelial or immune cells is governed by the NFκB pathway. NFκB is the key transcription factor mediating the inflammatory response at the intracellular level and controlling the production of cytokines in cells. One of the motivations to make use of mathematical modeling in the context of bacteria lung infection is that, both the cell-to-cell and intracellular levels contain feedback loops (see the examples). These loops are known to induce non-linear, counterintuitive dynamics, which requires quantitative data and mathematical modeling to be analyzed.

Bacteria and other microbes can invade the lung through the airways. When pathogens reach the lumen of lung alveoli they can replicate and attack the tissue using virulence factors, their own chemical weaponry (Figure 1). Upon recognition of pathogens, the immune response is initiated to clear them from the infected sites, and this process involves the secretion of cytokines and recruitment of immune cells.

A balanced immune response can be achieved via interacting immune cells that are controlled by intracellular regulatory networks of interacting molecules, such as cytokines, receptors, kinases, transcription factors, or non-coding RNAs. Such a system contains regulatory motifs, especially positive and negative feedback loops, which increase the complexity of the response and can provoke non-linear behaviors such as bistability and oscillation (Ref). For patients with respiratory bacterial infections, severe pathological condition can emerge if their immune systems fail to quickly neutralize the infection and to avoid systemic spread of the pathogen. On the other hand, overwhelming host immune response to the pathogens is also dangerous and can impede the proper functioning of the lung and other organs. So, any new treatments using the combination of antibiotics and immunomodulatory drugs will be useful if they can help the patients to maintain a balanced immune response (Wentker et al., 2017), which is governed by the multi-level biological system (Eberhardt et al., 2016).

**Figure 2:**
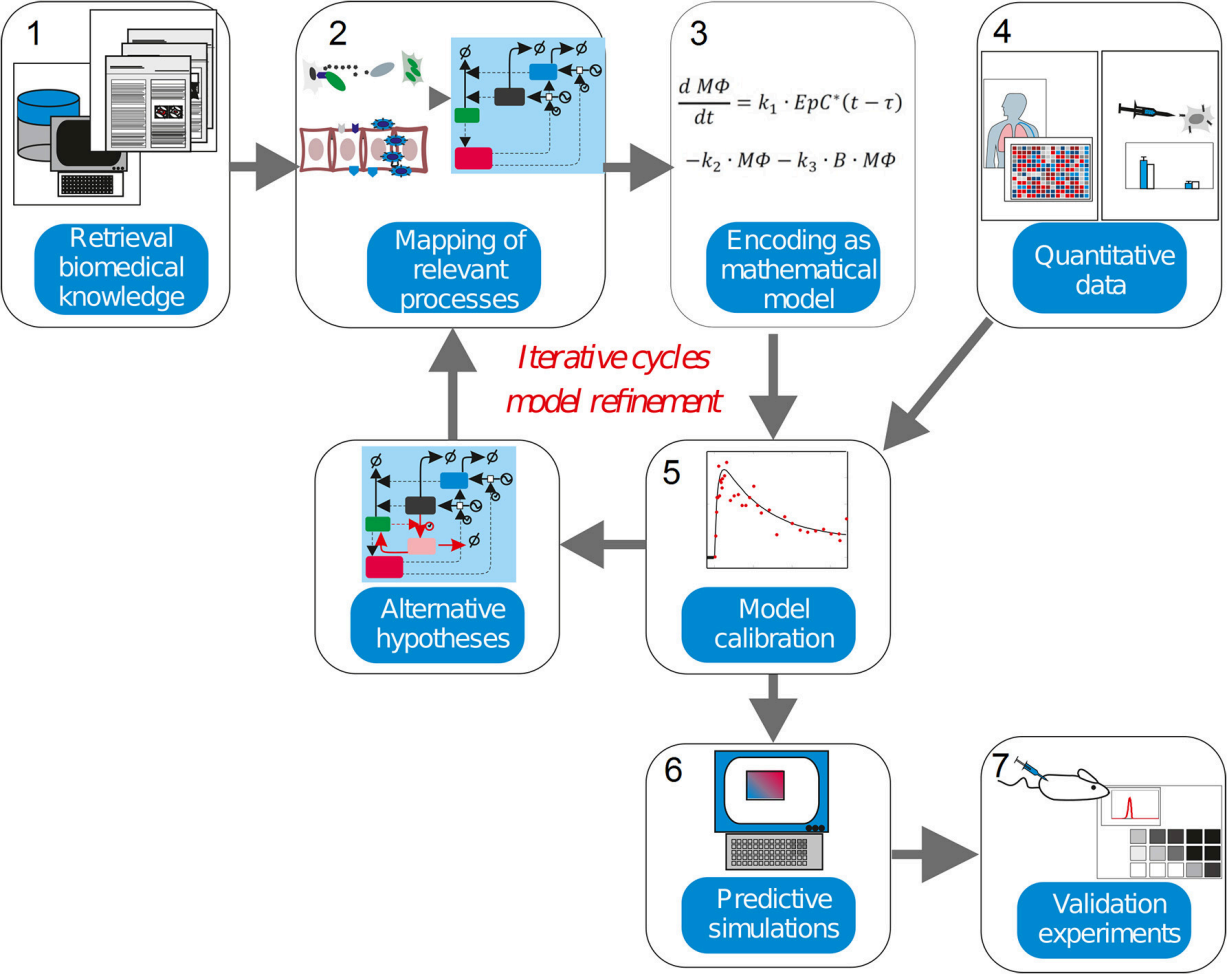
The systems medicine workflow. Systems biology modeling is about encoding biological knowledge into mathematical models whose simulations are used to dissect the molecular mechanisms behind diseases. Current biomedical knowledge is retrieved from publications and databases and used to apprehend the critical processes in the biomedical scenario investigated (1). These processes, the cell types, molecules and interactions involved are mapped into a graphical representation (2). Following some heuristic rules, this map is encoded as a mathematical model (3). For the characterization of the model in biological terms, quantitative experimental data is integrated into the model equations in a process named model calibration (4–5). The model is assessed to judge its ability to precisely reproduce the data available (6). An inadequate model leads to formulate alternative hypothesis and modify the model equations in accordance, thereby iterating the steps 3–5. (7). An adequate model is used to make simulations with predictive power, which generate new insights into the pathophysiology of the investigated disease once experimental validation is performed.

This level of complexity is equivalent to other natural and artificial systems, like those controlling large and modern aircrafts. For decades researchers in physics and engineering have been using mathematical modeling and simulations as an irreplaceable tool when trying to understand, predict or redesign these systems. Systems Medicine is the natural extension of this strategy to the biomedical domain. In our context, mathematical modeling can be used: (a) to inspect and integrate different but complementary types of quantitative experimental and clinical data, (b) to design experiments, (c) to elaborate, analyze and discuss hypotheses, (d) to perform model simulation-based predictions for the course of a disease, or (e) the feasibility of conventional, newly developed or personalized treatments (Vera and Wolkenhauer, 2008). For our purposes, Systems Medicine is a methodology that employs mathematical modeling to integrate and analyze quantitative biological data (Auffray et al., 2009; Wolkenhauer et al., 2013; Eberhardt et al., 2016; Figure 2). In the approach, biological knowledge is encoded into mathematical models whose simulations are used to dissect the cellular and molecular mechanisms behind diseases.

In a nutshell, the workflow is composed of several steps (Figure 2). The model derivation begins by **retrieval biomedical knowledge (1)**, biomedical information from publications and databases is used to identify the key compounds (cell types or molecules) and their interactions, and translated the information into a graphical depiction named regulatory map, **mapping of relevant processes (2)**. Based on the information gathered and some heuristic rules, this map is **encoded as a mathematical model (3)**, which consists of equations or other mathematical entities. In **model calibration (4-5)**, quantitative data obtained from experiments are used to characterize the mathematical model. This is often done though a computational process called “model calibration,” which assigns values to the parameters characterizing the model equations, such as the model becomes able to reproduce the existing quantitative data. Model calibration can often confirm or disprove the hypothesis encoded by the model equations. The inability of the mathematical model to reproduce the data leads to its reformulation, and eventually to the design of new experiments. In **predictive simulations (6)**, a calibrated model is used to generate new insights into the pathophysiology of the investigated disease via computer simulation. Finally, further **validation experiments (7)** are used to confirm or discard the predictions made via model simulation.

In the same manner as one cannot elucidate all the mysteries of modern biomedicine using a single experimental technique, say confocal microscopy, a single class of mathematical model among the plethora of those available in systems medicine is not useful for every purpose. Every problem or hypothesis to be explored requires a carefully selected and specific modeling approach. In this paper, we discuss and illustrate the distinctive features of different mathematical modeling frameworks with cases studies in the context of bacterial lung infection. Further, we compile and discuss relevant published results on the mathematical modeling of pathways and networks modulating the immune response, the host-pathogen interaction and the occurrence of coinfections, all of them topics relevant for bacterial lung infection. Finally, we discuss how to make use of this multiplicity of modeling approaches to open new avenues in the search of molecular and cellular insights in bacterial lung infection. This review is intended for modelers who want to enter the field of bacterial lung infection and need a review of published work, but also for infectiologists and immunologists interested on understanding how mathematical modeling can help them designing and interpreting their quantitative data and hypothesis. In the main text we focus on the basis of the modeling workflow, the modeling approaches and the published results, while further details in the methodologies discussed and the examples proposed are provided in Supplementary Material.

## Mathematical modeling of bacterial lung infection

In the context of lung infection, the use of mathematical modeling is especially suited because one is interested on elucidating the function and regulation of cell-to-cell or biochemical networks governing the local or systemic activation of the epithelial and immune cells in the course of lung bacterial infection. These networks are large and tightly interconnected; further, they display complex patterns of temporal activation. Moreover, one can be interested on integrating quantitative clinical and biological data accounting for the dynamics of the infection across different time- and spatial scales. Some events triggering the early local lung infection happens within minutes to hours, while the systemic phase of the immune response and the recovery and tissue repair can last days to weeks. Something similar happens at the spatial organization, with microscopy-level events like the triggering intracellular networks or the networks of interacting immune cells at the infection site, and mesoscopic-level events accounting for the effects of infection in the make-up and functioning of structures the lung alveoli and the airways. This level of complexity in terms of structure and data can be managed using different types of mathematical modeling. In the following we discuss in detail several modeling approaches, as well as the context during bacterial lung infection in which they are valid.

## Boolean models

### Main features of boolean models

**Figure 3:**
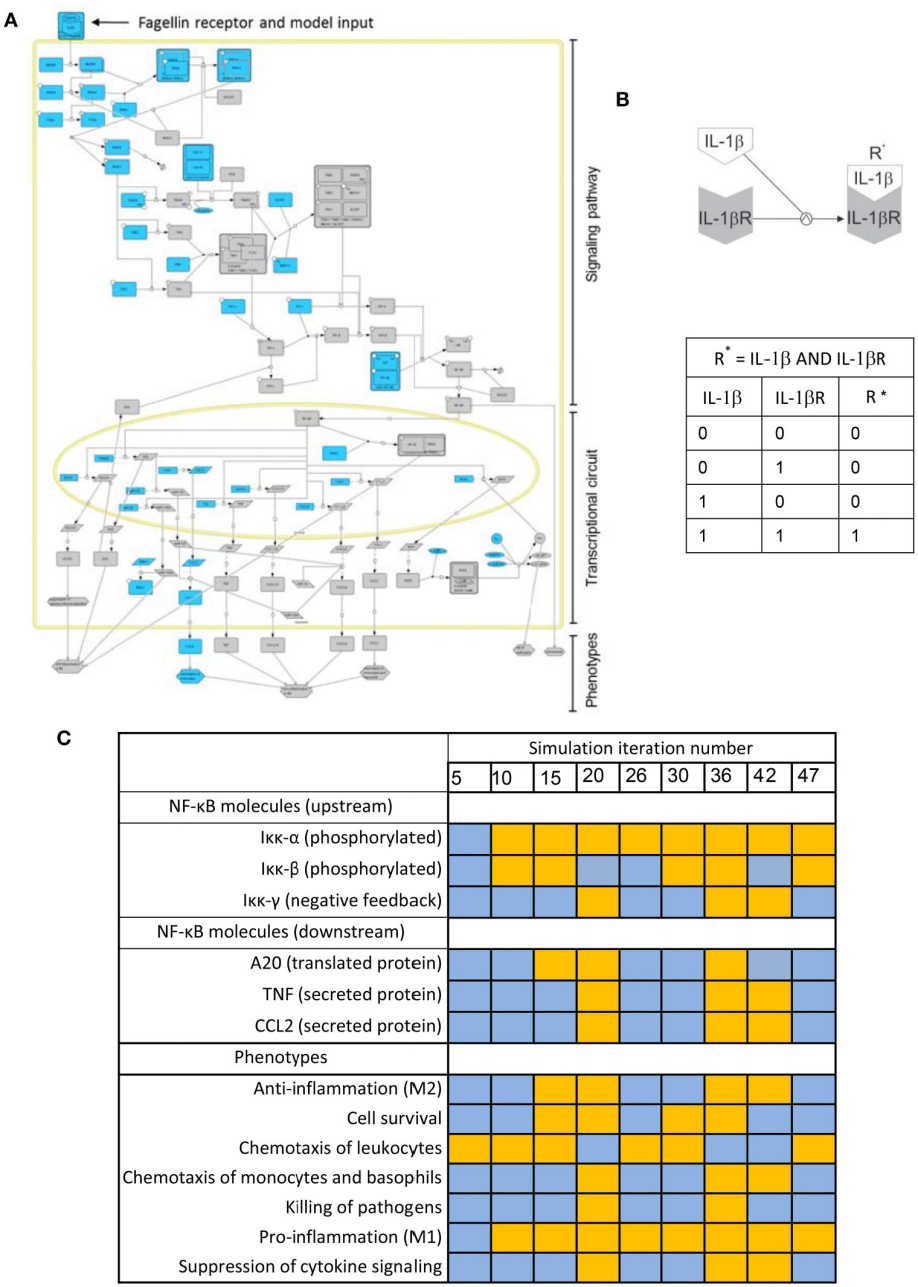
Boolean modeling of the NFκB pathway driving macrophage activation in bacterial lung infection. **(A)** Graphical depiction of the Boolean network. A full page visualization of the network is proposed in the Supplementary Material. **(B)** The activation of the IL-1β receptor (IL-1βR) upon binding of its ligand (IL-1β) modeled as an AND Boolean logic function. The table below the depiction represents all the possible combinations for the values of IL-1β and IL-1βR and their effect in the activation of the receptor (R^*^). **(C)** State of key nodes of the TLR5 macrophage network at different time iterations after igniting the input signal (flagellin = 1). Blue stands for nodes off (0) at the iteration considered, while orange indicates they are activated (1). These and other simulations can be visualized as animated gif files at http://sysbiomed-erlangen.weebly.com/resources.html.

Biochemical systems, if treated as networks of interacting entities, share many of the structural and regulatory features of electronic circuits. Boolean models, conceived for designing electronic circuits, were proposed 50 years ago as a tool to investigate the structure and dynamics of biochemical networks (Kauffman, 1969, 1993). For biochemical systems, Boolean networks are graphs in which nodes represent molecules and edges represent interactions between molecules. The interplay between molecules and biochemical reactions is represented using Boolean logic, i.e., discrete models in which every node or molecule can have only binary values: 0 or “OFF” (indicating the nonexistence or no-activation of the considered biochemical species), and 1 or “ON” (corresponding to its existence or activation). For example, Figure 3B is a depiction of the activation of the IL-1β receptor (IL-1βR) upon binding of its ligand (IL-1β). The process can be modeled using a Boolean logic function “AND.” The table in Figure 3C represents all the possible combinations for the values of IL-1β and IL-1βR and their effect in the values of the activated receptor IL-1βR^*^. One can see that activation (IL-1βR^*^ “1”) is only possible if IL-1βR and IL-1β are present (both with value “1”).

In Boolean Networks (BNs), the set of functions used to represent interactions is reduced to the basic logic gates “AND-OR-NOT” (for definition of logic gate and any blue marked word, see Glossary Section). However, logic gates can be combined in multiple ways and therefore complex multi-molecule interactions can be represented (Shmulevich and Aitchison, 2009; Wang et al., 2012). In line with this, the intracellular regulatory networks underlying the activation of immune cells can be investigated using Boolean modeling (Saez-Rodriguez et al., 2007; Kang et al., 2011). For example, Figure 3A is the graphical depiction of a Boolean network representing the triggering of NF-κB signaling, the master controller of the immune response, upon activation of Toll-like receptor 5 (TLR5). This event happens when the bacterial flagellum “is sensed” by the macrophage upon the binding of the bacterial protein *flagellin* to TLR5 (See Supplementary Material). The network is sequentially organized with the receptor activation as input, the subsequent activation of the NF-κB signaling pathway at the cell cytoplasm and the triggering of an NF-κB transcriptional circuit in the nucleus. In the network, nodes account for the network compounds, primarily different biomolecules like proteins and miRNAs, but also the cellular phenotypes triggered by the network. Further, the network edges account for the mutual interactions between the compounds, which are in the model represented like Boolean logic functions.

By combining and integrating this simple logic functions over the network in the course of a computational simulation, one can represent the complex sequential activation of the biochemical network modeled. A computational simulation is the mimicking the behavior of the system in a given biological scenario using the equivalent mathematical model: an *in silico* trigger emulates the correspondent biological signal, and the state of all elements of the model is updated at each iteration step by considering the state they assumed at the previous step, thus imitating the propagation of the signal throughout the network. The simulation considers discrete time points representing the activation state of the network, but the time between two consecutive time points (two steps of the simulation) is always assumed as uniform which does not necessarily reflect the expected biological time. Simulations can be used to predict the behavior of the system in non-tested experimental conditions. For example the Table in Figure 3C is a representation of the state of a few key nodes of the TLR5 macrophage network at different iterations in a simulation that mimics the triggering of the system after igniting the input signal (flagellin = 1). Blue stands for nodes off (0) at the time iteration considered, while orange indicates they are activated (1).

### Examples of boolean models in literature

In an interested case-study, Saez-Rodríguez et al. derived a large-scale Boolean network to represent the activation of T cells (Saez-Rodriguez et al., 2007; Kang et al., 2011). T cells, which belong to the adaptive branch of the immune response, can play a role in the long-term response to lung infection (Chen and Kolls, 2013). The network included the signaling pathways downstream of the T cell receptor, the CD4/CD8 co-receptors, and the accessory signaling receptor CD28. Altogether the network with 94 nodes and 123 interactions includes the primary mechanism behind the activation of T cells and depicts the complexity of biochemical pathways and the reciprocal crosstalk. Saez-Rodriguez et al. exploited one of the main advantages of Boolean networks in their analysis: Boolean models have very low computational requirements for simulation when compared with almost any other model and therefore they scale well with network size (i.e., they can simulate large networks). In line with this, they used their Boolean model to simulate and predict in a systematic and qualitative manner the effect of a large number of gene knockouts (“*in silico knockouts”)*. Based on the simulations, the model predicted that antibody-mediated perturbation of CD28 and the genetic knockout of the kinase Fyn, two of the network compounds, may have relevant effects on the network activation, and these effects could be validated experimentally. Using a strategy similar to that of *in silico knockouts*, Boolean networks have been used to predict the effect of drug combinatory treatments in cancer (Layek et al., 2011). We do not see any formal limitation impeding the use of the same strategy to predict the effect of the combination of antibiotics and immunomodulatory drugs in acute infections like bacterial pneumonia.

In line with this example but in the context of lung infection, Anderson et al. (2016) studied human dendritic cell response against the influenza H1N1, a virus that can co-infect with several types of bacteria to produce pneumonia (Joseph et al., 2013). To this end, they derived a biochemical network with 13 nodes corresponding to genes and transcription factors playing a role in antiviral response (e.g., NF-κB, STAT1 and IRF1), and 42 edges representing the activation of key immune pathways during the infection. The simulations were done with an asynchronous Boolean model. The initial states of the Boolean simulations were based on experimentally observed expression patterns for the genes in the network (e.g., EGF, NFAT, PDGF and IL-2 set as active during H1N1 virus infection). The model was used to investigate the regulation of the IL-2 pathway after exposure to influenza virus. The model simulations suggested that NFAT can regulate IL-2 signaling in the context of the virus infection, a prediction that was experimentally validated. Further analysis led to the conclusion that IRF and NK-κB signaling share regulatory functions in H1N1, two out of the three major signaling pathways responsible for mediating TLR-induced responses in viruses, bacteria and other pathogens (Mogensen, 2009).

Although Boolean models are more suited for investigating biochemical networks, they can also be used for describing networks of interacting cell population's exceptions (Jack et al., 2011). For example, Thakar et al. (2007, 2009, 2012) developed a Boolean model for the regulation of the immune system response during the respiratory inflammation caused in mouse by two close relatives of the Bordetellae genus: *Bordetella bronchiseptica*, a bacterium causing infectious bronchitis in animals, and the human pathogen *B. pertussis*. The model contains well-established knowledge on the immune response after independent infection with each one of the bacterium. The nodes represent (a) immune cell types involved in inflammatory process, including dendritic, T or B cells, (b) cytokines related to a specific phase of the immune response or (c) antibodies. Some edges account for the activation of the immune cells upon stimulation, while others connect the active immune cells to the production and secretion of cytokines and antibodies. Thus, a Boolean network can be used to integrate cell-to-cell and intracellular scale events. In the network, synchronous and asynchronous simulations were performed with the Boolean model. Further experimental data on the host- and pathogen interaction were used to refine the logic gates describing the behavior of the nodes. Model simulations identified three phases in the course of the *B. bronchiseptica* induced inflammation, and suggested that antigen regulatory mechanisms play a prominent role along the whole process, conclusions that were experimentally validated.

In a continued work, the model was expanded by including published experimental data on time evolution for the concentration of IL-10 and IFNγ, information useful to expand the network by including the differentiation of naïve T cells. Model simulations proved to be able to make several predictions namely: (1) the cooperativity between IL-10 and IL-4 signaling to inhibit INFγ, which was later experimentally validated; (2) the role of the interactions among IL-10, INFγ, IL-12, and IL-4 signaling in deciding the naïve T cell differentiation process into either Th1 or Th2; and (3) the fact that Th1 cell activity must be temporally longer than that of Th2 cells. To integrate time-series data, the authors transformed the discrete model into a hybrid model (see final section of this paper). Further, the group adapted the network to investigate the co-infection of rabbits with *B. bronchiseptica* and *Trichostrongylus retortaeformis*, a worm that usually infects herbivores inducing a severe infection. Helminth infections predispose mice to pneumococcal pneumonia (Apiwattanakul et al., 2014), and some helminths can trigger pneumonia in humans (Cheepsattayakorn and Cheepsattayakorn, 2014). Using previously published experimental data describing the host immune response to the single infection and for co-infections, an asynchronous Boolean model was derived. Boolean logic functions were derived from literature, and in case of uncertainties, functions were adjusted by comparing the simulation output with experimental results. The resulting Boolean model was used to investigate the crosstalk between regulatory pathways upon the infection with the two pathogens. To validate the co-transfection network, the group infected rabbits with both pathogens, and then assessed the robustness of the model by comparing the resulting activation pattern of the immune response network upon infection with data obtained in rabbit model. Further, simulations representing single knockout of selected network compounds were used to determine central nodes of the single and co-infection networks, with special attention to the knockout of cytokines and immune cell populations' nodes. For example, knockout of nodes accounting for populations of B, dendritic or T cells led to a longer persistence of the bacteria in all case studies. In contrast, knockout for the IL12II or eosinophil population nodes in the co-infection network rendered parasite population not persistent anymore.

### Critical remarks on boolean models

There are some alternative modeling frameworks derived from Boolean logic. For example Probabilistic Boolean Networks (PBNs) use Boolean logic and Boolean values, and then implement a set of probabilistic rules determining the state of each node. Each rule is associated to a probability that a specific network state can occur based on the states of its inputs, and the probability for the transition can be assigned based on experimental data (Shmulevich et al., 2002). This probabilistic feature can make PBNs interesting to account for immune cell interactions with a probabilistic compound due to the low abundance of the cells involved at the site of interaction, but also for intracellular interactions with molecules in low abundance (Celli et al., 2012).

Despite the complexity of the interactions that can be modeled by pure or probabilistic Boolean logic, the universe of possible values for every network node is always reduced to 0 and 1. In multi-valued logic models each node can assume several discrete values that refer to a specific qualitative property (for example, “0” for no significant amount, “1” for small amount, and “2” for large amount of receptors activated). This approach has proved to be very valuable in some cases like transcriptional activation. For example, one can have transcriptional targets requiring low levels of active NF-κB, while others may require much higher levels, and a multi-valued model may be able to account for this distinctive activation pattern. In these models, thresholds can be set to determine the qualitative behavior of the node (Schlatter et al., 2009; Guebel et al., 2012).

Further, Boolean networks can be “calibrated.” This calibration is named pruning and consist on a systematic addition or deleting or nodes or interactions based on the use of quantitative data. In this way, one can make use of -omics data sets to refine the structure of the Boolean network (Terfve et al., 2015). Boolean networks are not suited for spatial features associated to biochemical reactions like molecular gradients. But perhaps the main limitation of Boolean models is their poor ability to reproduce and simulate the non-linearity arising from the existence of regulatory loops in biochemical networks. In consequence, they cannot provide detailed analysis of the fine-tuned regulation of biochemical systems enriched in these motifs. Mathematical models that can handle successfully nonlinearity are those in ordinary differential equations, which are discussed in the context of lung infection and inflammation in the coming section.

## Models in ordinary differential equations

### Main features of ODE

Under the assumptions that the biochemical reactions happen in discrete and homogenous intracellular regions (Rahmandad and Sterman, 2008) and the velocities of the biochemical reactions are determined by the concentration of the intervening species (Gustafsson and Sternad, 2013), biochemical networks can be modeled using kinetic models. Kinetic models are a special type of models in ordinary differential equations (ODE), where the equations describe the rate of change of the populations of the biomolecules involved in the biochemical reactions. Similar types of ODE models can be derived to account for the dynamics of interacting cell populations.

**Figure 4:**
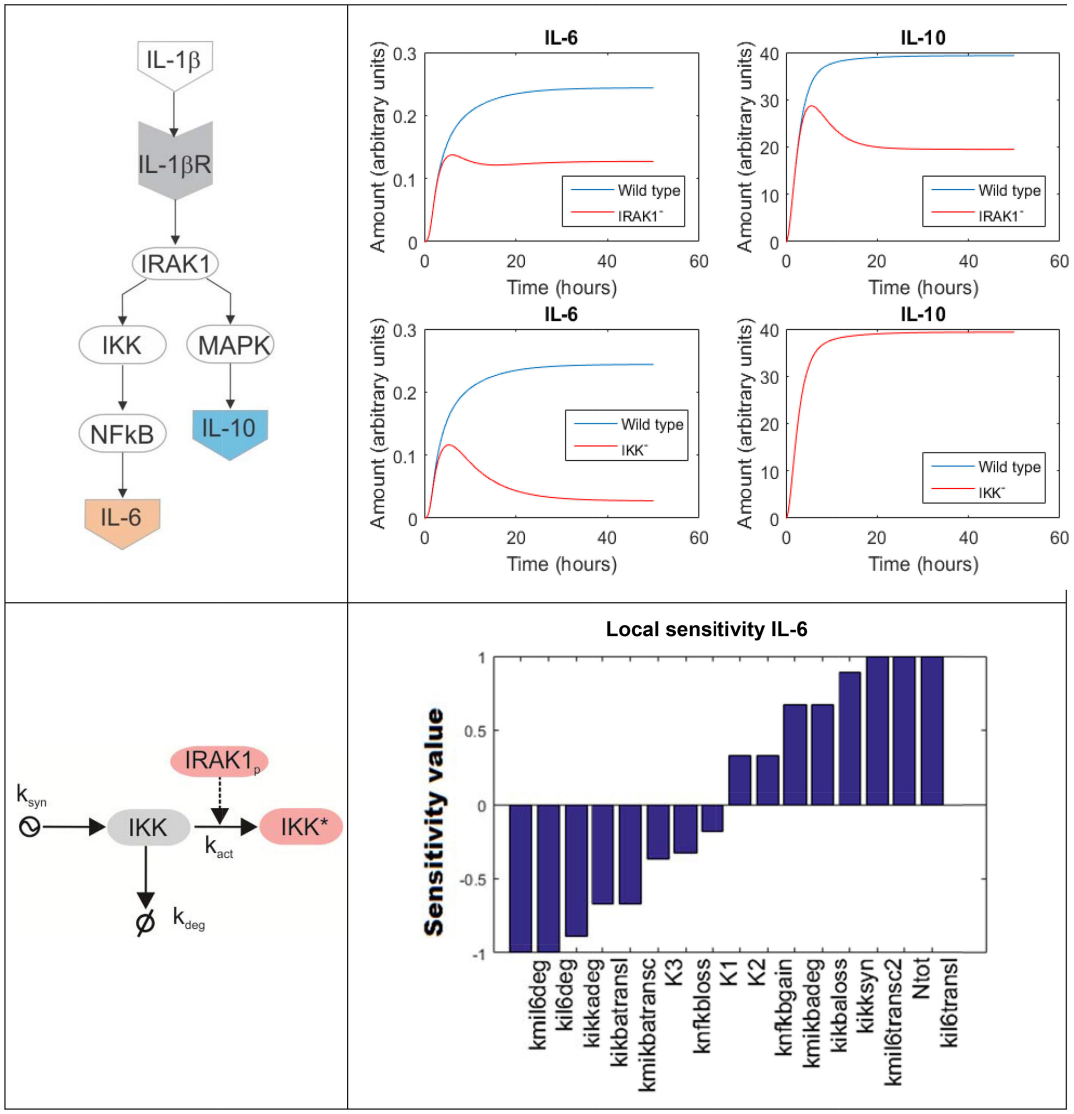
ODE model accounting for the inflammatory response triggered in lung epithelial cells upon activation of the IL-1β receptor. **Top left:** simplified depiction of the two branches of the inflammatory response triggered upon activation of the IL-1β receptor included in the model. **Top right:** model simulations accounting for the production of IL-6 and IL-10 in response of IL-1β mediated NF-κB activation under IRAK1 (**Top**) and IKK (**Bottom**) knockout conditions. **Bottom left:** detailed scheme of the accounting for the processes affecting in the model the values of inactive IKK. The arrow finishing in null symbol accounts for degradation of IKK, while the arrow starting in a ying-ying symbol stand for synthesis. **Bottom right:** local sensitivities of IL-6 concentration with respect to the perturbation of the model parameters (see Supplementary Material For further details).

To model a biochemical network composed by several molecules, one has to formulate a system of coupled differential equations, consisting on one equation per each element of the system whose dynamics is modeled. For example, Figure 4 top left is a simplified depiction of an ODE model accounting for two branches of the inflammatory response triggered upon activation of the IL-1β receptor by its ligand in lung epithelial cells (a detailed scheme can be found in Supplementary Material). One branch mediated by NF-κB promotes the secretion of several pro-inflammatory cytokines like IL-6, while a second branch mediated by MAPKs promotes the secretion of, among others, the anti-inflammatory cytokine IL-10. The branching point is the activation of IRAK1. The model accounts for the changes on time of the network compounds using the mass-action formalism. For example, the following equation represents the rate of change of the concentration of inactive IKK (IKK, see Figure 4 bottom left):

$$\begin{array}{l} \frac{\text{dIKK}}{\text{dt}}=k_{syn}-{\text{k}}_{act}\cdot \text{IKK}\cdot {\text{IRAKI}}_{\text{p}}-k_{deg}\cdot \text{IKK} \end{array}$$

In the right-hand side of the equation, each term accounts for a process affecting the concentration of IKK. The first term represents the synthesis of IKK, here modeled like a process at constant (mM·h^−1^ units), stable functioning and represented by the parameter *k*_*syn*_ The second term accounts for the phosphorylation and activation of IKK, represented by a rate equation proportional to the quantities of inactive IKK and phosphorylated IRAK1 (IRAK1p) and multiplied by a rate constant (${\text{k}}_{act},\text{m}{\text{M}}^{-1}\cdot {\text{h}}^{-1}\text{unit}$). The third term models the degradation of inactive IKK, which linearly depends on its concentration and the rate parameter *k*_*deg*_ (h^−1^ units).

Contrary to Boolean networks, ODE models can be used to make continuous and precise time-depending simulations. For example, one can simulate the effect of deactivating mutations in key genes of the NF-κB pathway in the secretion of cytokines by lung epithelial cells. Figure 4 top right displays a set of predictive simulations accounting for the production of IL-6 and IL-10 in response of IL-1β mediated NF-κB activation under knockout conditions. The wild type condition is displayed in blue. In addition we showed the predicted time profile for both cytokines under deactivating mutations of IRAK1 (here represented as IRAK1^−^) and IKK (IKK^−^). Compared to Boolean networks, the simulations are continuous, more detailed and give quantitative information about the duration and the intensity of the cytokine secretion in the different conditions simulated. For example, the model simulations indicate that the IRAK1 mutation (IRAK1^−^) has a significant effect in the production of IL-10 and can lead to a 50% decrease in its maximal concentration. Similarly, IKK mutation (IKK^−^) reduces the secretion of IL-6 while not affecting IL-10. These model predictions match published experiment reports (Supplementary Material).

ODE modeling is a well-established methodology in biomedicine, often included in the training offered in master programs in computer sciences, physics, computational biology and bioinformatics. The key feature of ODE models is the existence of a large array of computational and theoretical techniques of model analysis beyond simple simulations. This includes sensitivity analysis (Savageau, 1971; Zi, 2011; Castillo-Montiel et al., 2015), symbolic analysis (Ibargüen-Mondragón et al., 2014), bifurcation analysis (Duan et al., 2011; Yuri, 2017), design space analysis (Savageau, 2011) model optimization (Vera et al., 2003; Zhang et al., 2015) and parameter estimation and identifiability (Raue et al., 2009).

For example, in sensitivity analysis one can obtain quantitative information on how variation in the value of given model parameters can affect the dynamics and values of the model's time-dependent variables (Saltelli et al., 2000). In our example (Figure 4), we focus on local sensitivities, which are calculated in a narrow region of the model parameter values around the condition of interest, though it is possible to perform sensitivity analysis for a wider interval using global sensitivities (Mathew et al., 2014). In our case, local sensitivity analysis allows for detecting the model parameters affecting the most the maximal value of IL-6 during the simulation, here used as a measure of the production of pro-inflammatory cytokines in the course of cell activation. We computed the local sensitivities by varying the parameter values within a small interval around its value (the value of the parameter set was arbitrarily defined in a way it resulted biologically feasible and instructive for the purpose of this review). The perturbed parameters are ordered in terms of their effect in the maximal value of IL-6, from those whose increase negatively affects IL-6 to those that make a positive effect (Figure 4 bottom right). In a real case-study, the output of this analysis could be used to select promising molecular drug targets for new immune modulatory drugs. These drugs could be administered in parallel to antibiotics and would modulate the production of pro-inflammatory cytokines during the acute phase of the inflammation. A similar approach relying on ODE models and sensitivity analysis has been successfully utilized in anticancer drugs therapy (Schoeberl et al., 2009), and there are no evident limitations to make something similar in bacterial lung infection.

ODE models can account for highly non-linear processes and show properties, often found in biological regulatory circuits, like bistability or oscillation (Tyson et al., 2003). In that case, advanced model analysis tools can be used with ODE models to dissect the non-linear dynamics of inflammatory and infectious diseases. For example, ODEs can be combined with bifurcation analysis. In bifurcation analysis, advanced methods from non-linear dynamics mathematics are used to detect model parameters associated to key interactions and processes, for which its perturbation in given intervals generate a shift in the equilibrium of the system. Here, we are not talking about smooth, gradual changes like those detected by local sensitivity analysis, but about drastic changes such as those generated by the sudden activation of, for example, the positive feedback circuits behind several known autocrine loops in inflammation (Coward et al., 2002). Dunster et al. (2014) employed bifurcation analysis of an ODE model to analyze the role of different immune cells on the resolution of inflammation. The model accounted for the interactions between macrophages, neutrophils and pro-inflammatory mediators like Tα and IL-8. The model analysis focused on finding the bistability region in the model, that is, the set of model configurations in which the system can switch between the two physiological states: inflammation and resting. Based on their analysis, they concluded that key processes accelerating the resolution of inflammation are an increase of macrophage phagocytosis and the neutrophil apoptosis.

Moreover, these ODE model analysis methods can be integrated in workflows to investigate complex properties of biological systems (Nikolov et al., 2010). A very recent work created and analyzed a mathematical model of the *Streptococcus pneumoniae* lung infection (Domínguez-Hüttinger et al., 2017). It includes the interactions between the pathogen and the host like macrophages and neutrophils activation, bacteria clearance, epithelial cell barrier integrity and bacteria migration through the barrier to the vessels. In the model, the authors differentiated between a commensal state, that does not produce a disease, and an invasive and infective state of the bacteria. By including this feature in the bacteria population dynamics, the model predicted four different possible phenotypes: (i) sepsis, that is systemic bacteria spread and inflammation, (ii) immunological scarring, that is, cumulative, long-lasting immune response to pathogens inducing tissue remodeling and altered immune responses to new pathogenic challenges; (iii) sepsis + immunological scarring, or (iv) healthy infection recovery. Further, model simulations were used assess the required duration of antibiotic treatment to treat each phenotype.

Sparse information taken from the literature can be used to characterize model parameters. Based on predictive model simulations using the data-based model, one can gain new insights on the regulation of the network underlying, for example, pathogen associated tissue destruction. The immune system residing in the respiratory mucosa has to achieve a balance between its ability to deplete pathogens and to induce tissue damage; a failure in this tightly control mechanism can induce chronic inflammation and tissue destruction (Lugade et al., 2011). Lo and coworkers (Lo et al., 2013) constructed and characterized with available data a model accounting for the abnormal regulation of T helper 1 (Th1), T cell helper 2 (Th2), and T regulatory cells (Treg) in chronic lung mucosal inflammation. The model was used to simulate possible physiological scenarios concerning inflammation of the lung mucosa. Based on the model simulations, the authors found that deregulation of the interaction between these immune cells is sufficient to explain the emergence of chronic lung mucosa inflammation. Specifically, the model predicts that upon Treg downregulation the Th1 and Th2 responses to cytokine can be abnormally high. Since it is known that mucosal Th1 and Th2 cells can produce pro-inflammatory cytokines (Neurath et al., 2002), the system displays the structure of an autocrine positive feedback loop, which could induce under deregulation signal amplification and chronic inflammation.

In predisposed patients, airways and lung infections caused by both viruses and bacteria can unbalance the regulation of the local lung immune system and contribute to asthma exacerbation (Pelaia et al., 2006). Chernyavsky et al. (2014) derived an ODE model on the emergence of airway smooth muscle cells (ASMC) hyperplasia due to asthma-related inflammation, which was characterized using published data from biopsies and inflammatory biomarkers (Contoli et al., 2010). The authors modeled interactions between proliferative and non-proliferative ASMCs and their impact on the inflammatory state of the lung. The model was utilized to simulate the development of the asthma associated inflammation. Model simulations showed that the speed of inflammation resolution is a leading factor in the long-term evolution of asthma, and also that the features of the tissue remodeling during and after the inflammation are important to control the long-term evolution of asthma.

The parameters of ODE models can be estimated by fitting the model simulations to dense time series of experimental data in a process called model calibration. For example, Mochan et al. (2014) modeled pneumococcal lung infection and used time series data from tittered mice infection to calibrate the model. Bacteria titration refers to the inoculation of different initial amounts of bacteria to mice. The model included the interplay between the bacteria, lung epithelial cells and alveolar macrophages, the production of cytokines and chemokines and the subsequent recruitment and activation of neutrophils and monocytes. The model was used to simulate and quantify the dynamics of the damage in the tissue caused by the immune system in the early phases of infection. The model simulation analysis pointed to the importance of the dynamics of macrophage phagocytosis to explain the differences between the phenotypes of resistance or sensitivity to pathogen. In a different work, Guo et al. (2011) integrated time series data of bacterial burden in an ODE model to quantify the contribution of neutrophils on the bacterial clearance during pneumonia in mice. To this end, the authors formulated a single-equation model accounting for the dynamics of bacterial growth when exposed to lung neutrophils. The model not only correctly predicted the number of neutrophils that is necessary for suppressing *A. baumannii* growth by 50%, but it also proved to be able to make predictions for the case of infection with other pathogens like *P. aeruginosa*.

### Examples of ODE models in literature

Smith et al. (2011) built a model accounting for the role of resident alveolar macrophages, neutrophils and monocyte-derived macrophages in early lung infection by *S. pneumoniae* in mice. The model includes time-dependent variables for the bacteria population, resting and active macrophages, activated and non-activated epithelial cells, cytokines, neutrophils and the debris associated to infection and tissue damage. To assign value to the model parameters they extracted information from literature, but also fitted their model to time series data for different bacterial titration. The model was used to quantify the contributions of cytotoxicity and immune-mediated damage in pneumococcal pathogenesis. When the authors generated two alternative versions of the model with or without monocyte-derived macrophages recruitment, the dynamics of bacteria growth was not affected. Based on the previous work, Schirm and coworkers proposed a modified mathematical model of cellular interactions in bacterial pneumonia (Schirm et al., 2016). They considered in the model alveolar macrophages, neutrophils and monocyte-derived macrophages. This model was fitted with large time-series data sets from infected mice, which includes measurements for pneumococci, neutrophils and macrophage populations, as well as for IL-6 and debris, here assimilated to histological damage score measured in the lung tissue. The calibrated model was used to simulate the evolution of the disease with or without antibiotics treatment. To this end, the model simulated the administration of 0.02 mg/g Ampicillin or 0.1 mg/g Moxifloxacin every 12 h, starting 24 h after infection. The model simulations indicate that alveolar macrophages are responsible for the quick elimination of the disease. Moreover, the model simulations predicted that the remission of the infection can happen with lower doses of antibiotics than those applied in the experiment. In line with this, the authors propose to utilize model simulations to design alternative time schedules for the antibiotic treatment. This strategy could be relevant in the context of bacterial infection induced sepsis. Sepsis is a common cause of acute kidney injury and therefore a modeling-based methodology for accurate antibiotics dosing could be relevant for critically ill patients (Eyler et al., 2011). To this end, one can derive a pharmaco-kinetics and pharmaco-dynamics ODE model accounting for the toxicity and effectiveness of antibiotics, similar to existing models accounting for efficacy vs. toxicity of anticancer drugs (Ballesta et al., 2011).

Coinfections, the co-occurrence and potential synergy between two infectious agents, have been also investigated with ODE models. An example of modeling coinfection is the work by Smith et al. (2013), in which coinfection of mice with influenza virus and *S. pneumoniae* in the lung was investigated. The model includes variables accounting for the dynamics of influenza virus, *S. pneumoniae*, alveolar macrophages and influenza lung epithelial target cells. The model was calibrated using time-series data for the amount of bacteria and virus. Remarkably, the model simulations showed the rebounding in the populations of the bacteria and the virus. Pathogen rebounding is the proliferation of a pathogen after an initial decrease when it co-occurs with a second pathogen. In Smith et al (Domínguez-Hüttinger et al., 2017), upon infection with bacteria, the virus population rebounds due to the release of viruses that were latent in the immune and lung cells killed by the bacteria. In parallel, the model predicts an increase in the bacterial load due to the impairment of macrophage response provoked by the presence of the viruses. The system as described displays the structure of a positive feedback loop in which bacterial and virus infection amplify each other.

### Critical remarks on ODE models

ODE models can account for important spatial features like molecular gradients only in a very limited manner. An extension of ODE models in this regard could be partial differential equations (PDE) models; however, the lack of appropriate experimental data for their characterization has limited their development in biology to a few but promising case studies (Matzavinos et al., 2004; Murano et al., 2014). In an ideal setup, ODE models require numerous and rich time series data sets for model calibration, a prerequisite to obtain a trustable model. This necessity for complex data sets is a clear limitation, especially when trying to model large biochemical networks. A fundamental limitation of ODE crucial for some biological systems and transcriptional circuits is that predictions based on ODE models may fail for systems with low copy numbers for the molecules or the cells involved in the interactions, in which randomness in their dynamical behavior emerges. These special features are better represented by stochastic models, which are discussed in the coming section.

## Stochastic models

### Main features of stochastic models

**Figure 5:**
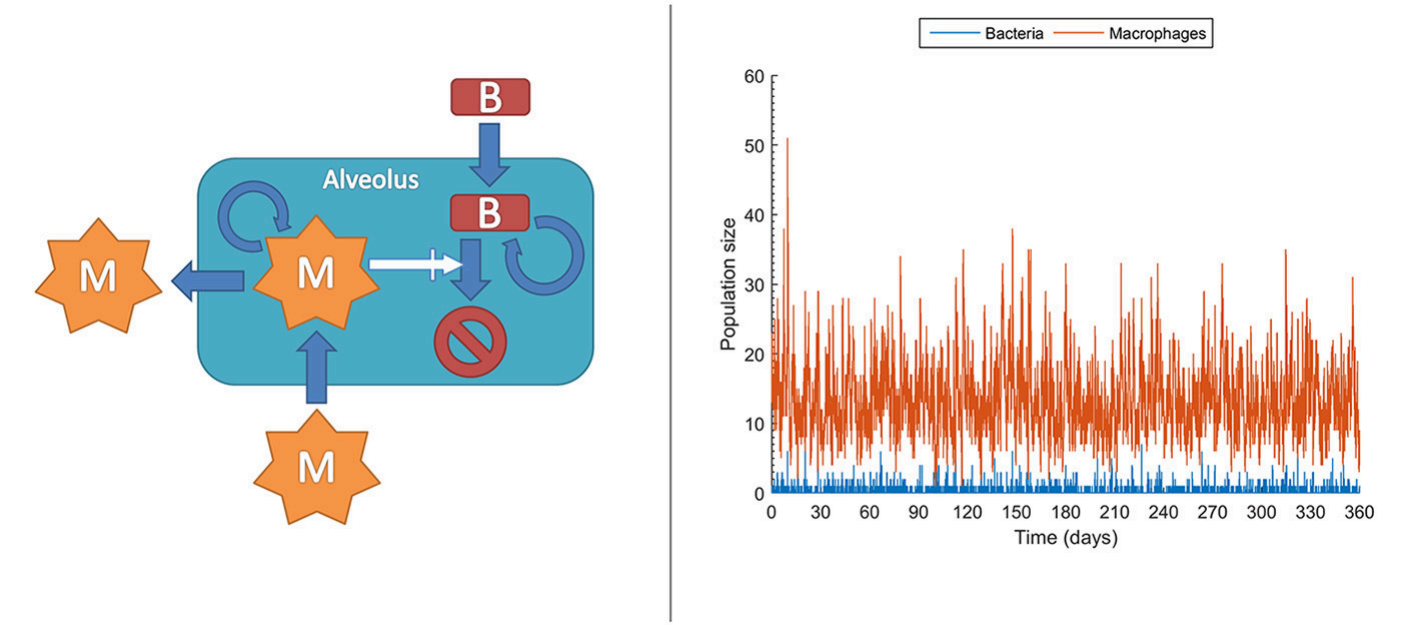
Stochastic model accounting for the dynamics of infection of an alveolus with low but prolonged exposure to bacteria. **Left:** sketch of the stochastic model. M, macrophages; B, bacteria. **Right:** Single realization of a stochastic simulation for bacteria and macrophages populations (see Supplementary Material for more details).

At the molecular level, chemical events, including biochemical reactions, occur randomly. Taking this strong assumption, it is impossible to deterministically predict when the next reaction occurs, but also each experimental repetition of a biochemical reaction will intrinsically differ in the measured values. This effect is actually important under low copy numbers for the molecules intervening in the reaction, conditions under which it is known and it has been experimentally confirmed that accuracy collapses for deterministic models like those in ODEs. In contrast, stochastic models can account for this effect rather than attributing it to measurement errors, thereby outperforming deterministic models (Gillespie, 1992; Klipp et al., 2009; Pahle, 2009; Wilkinson, 2009; Ullah and Wolkenhauer, 2010). In stochastic models, chemical species or cell populations are represented as discrete random variables. These variables form the state space of the stochastic model and describe the abundance of each species at any given time point. Chemical reactions or cell interactions are envisioned as random processes that change the abundance of the involved species. While these reactions occur randomly, their probability of occurrence depends on the current state and it changes as the system moves from state to state. For example, in the very early phases, both bacteria and macrophages display very low copy numbers, sometimes with single macrophages patrolling one or more alveolus. In these conditions, even small random fluctuations can have a large impact on the population dynamics and therefore a stochastic model is an option for describing their population dynamics. Figure 5 left displays the structure for a stochastic model, adapted from Van Furth (2012), accounting for the long time dynamics of infection of an alveolus exposed to stochastic bacteria colonization. In the model, the current number of macrophages and bacteria is denoted by *m* and *b* respectively. The interactions between macrophages and bacteria determine the state transitions, that is, the increase or decrease of the bacteria and macrophage populations. For example, the stochastic model accounts for the generation of a macrophage (*a*_*M*+_) with the following equation:

(1) $$\begin{array}{r} a_{M+}\left(m,b\right)=c_{Mmigrate}+{c_{Mbirth}}^{*}m+{c_{Mresponse}}^{*}b^{*}m \end{array}$$

Here, is it assumed that the generation of a macrophage can occur in three different ways: (i) macrophage migration into an alveolus occurring at a constant probability rate (*c*_*Mmigrate*_); and (iii) recruitment of additional macrophages depending on the current number of bacteria and macrophages (${c_{Mresponse}}^{*}b^{*}m$). Figure 5 right is a single long time simulation of the model. The single simulation reveals large variability in the populations of bacteria and macrophages. In particular, the macrophage population shows large fluctuations, with values ranging from one to up to 51 macrophages in the alveolus, in conditions with very small amount of bacteria. When one performs a large amount of similar simulations (here 10^4^ simulations) one can verify that these fluctuations render the fate of the system stochastic. Thus, in a small fraction of the simulations (0.1%) the population of bacteria gets higher than 100. The stochastic model simulations suggest that, under healthy conditions and for low long term lung alveolus exposure to bacteria, most of the episodes of bacteria colonization are quickly resolved, although there is still a small probability of bacterial infection.

As discussed before, the core regulatory pathway controlling the activation after bacterial lung infection of epithelial and immune cells is the NF-κB pathway. There are two features that make stochastic modeling suitable for investigating NF-κB activation. Stochastic models are especially suited for transcriptional circuits because gene expression is widely considered to be a process dominated by randomness (Elowitz et al., 2002; Kaern et al., 2005; Wilkinson, 2009; Bressloff, 2017). NF-κB is a transcription factor, and under some conditions the pathway activation may lead to a low amount of transcriptionally active NF-κB molecules. In this case, large fluctuations may appear in the transcription of NF-κB targets, making advisable the use of stochastic modeling. In line with this and using a microfluidic cell culture platform and single cells resolution, Tay and collaborators investigated the features of NF-κB activation for a wide range of values of concentration for TNFα, one of the infection-associated ligands promoting NF-κB activation. Under low TNFα concentration, they found single cell heterogeneity and digital response of the cells. This translates into and all-or-none activation pattern for 3–50% of the cells at concentrations as low as 0.1–0.01 ng/ml. To elucidate the regulatory features inducing this behavior, the authors derived a stochastic model accounting for the NF-κB activation. Using the model, they found that the ability of the model to reproduce the digital response observed relied in the inclusion in the model equations of specific features of TNFα ligand and receptor turnover. Precisely, they found it was related to the limited TNFα amount present in the microfluidic chambers, the TNFα degradation and turnover and the cell-to-cell variability in the amount of TNFα receptor available for activation. Further, to reproduce the data the model assumed a non-linear nature to the IKK activation profile, attributed to the fact that IKK subunits IKK-α and IKK-β achieved full activity when phosphorylated at two different residues (Tay et al., 2010).

In addition, stochastic models are suitable for assessing the fine regulation of feedback loop circuits displaying oscillations or bistability because stochastic models can assess their sensitivity to small random perturbations (Levine et al., 2013; Dobrzyński et al., 2014). NF-κB signaling is controlled by a combination of intracellular negative feedback loops, which are able to induce oscillations (Nelson et al., 2004), and autocrine positive feedback loops with the ability to trigger bistable switches (Pękalski et al., 2013). In both cases, stochastic modeling is the right tool for assessing the sensitivity of NF-κB signaling to small random perturbations induced by these regulatory loops. Ashall et al. combined single-cell life imaging and modeling to investigate the role of these oscillations. They could show that the expression of a number of NF-κB transcriptional targets depends on the frequency of the potentially pulsatile inflammatory signals found at the site of inflammation and infection. Although these features could be investigated by ODE modeling, the heterogeneity of single-cell responses they found exceeded the capabilities of these models. However, a stochastic model that assumed delayed stochastic transcription for IκBα and stochastic transcription of IκBα and A20 (all of them inhibitors of NF-kB signaling embedded in negative feedback loops) proved to be able to recapitulate the cell-to-cell heterogeneity in the NF-κB oscillations. In line with these results, the same team recently showed the existence of single cell NF-κB-mediated oscillatory responses even under physiological concentrations of TNFα, a cytokine that play a pivotal role in the pathogenesis of pneumococcal pneumonia (Takashima et al., 1997; Ashall et al., 2009; Turner et al., 2010).

Other immune related intracellular pathways may display the features that make necessary the use of stochastic modeling. For example, intra- and extra-cellular calcium signaling plays an important role in the immune response (Vig and Kinet, 2009) and they have been described using stochastic models (Rüdiger, 2014). Further, TRAIL-mediated apoptosis, a mechanism playing a role in limiting the effect of alveolar macrophages on the extension of inflammation during *S. pneumoniae* lung infection (Steinwede et al., 2012), can display stochastic cell-to-cell variability in its activation (Bertaux et al., 2014). The dynamics of pathogenic bacteria intracellular circuits can become also stochastic (Norman et al., 2015). In line with this, Tuchscherr et al. (2011) showed that as part of their immune scape strategies, *Staphylococcus aureus* can induce a phenotype switching. Bacteria switching is a transient bacteria phenotypic change, governed by intrinsic stochasticity intracellular circuits, that provides bacteria with functional diversity and fast adaptation to environmental changes.

### Examples of stochastic models in literature

Stochastic models have been used for decades to dissect the cell population dynamics during lung infection. Two recent papers deal with the lung infection by *Francisella tularensis* (Gillard et al., 2014; Wood et al., 2014), an infectious intracellular gram-negative bacterium that infects primarily macrophages. When inhaled in an aerosol, *F. tularensis* can proliferate in the lung causing a type of severe pneumonia called pneumonic tularemia. Gillard et al. (2014) derived a stochastic mathematical model accounting for the early phases of *F. tularensis* pathogenesis in the lung. The model contained three possible states for the alveolar macrophages, coinciding with three of its most prominent phenotypes: (1) resting macrophages, functional but with no ability to kill bacteria; (2) suppressed macrophages, unable to overcome cytokine production and bacteria phagocytosis; and (3) classically activated macrophages, which play a role in clearing the infection. Regarding the dynamics of macrophages, the model considers as key events in the early infection phase the macrophage infection, its suppression and activation and death. Concerning the bacteria dynamics, the model accounts for bacterial proliferation, death and phagosome escape to the cytosol. To derive the model, the authors extended the framework of the **birth-and-death processes stochastic models** by attributing to each macrophage four features (spatial location, state of activation, number of phagosome bacteria, number of cytosolic bacteria) and making them affect the macrophage and bacteria populations dynamics (Levy and Green, 1968; Tranquillo et al., 1989). The model was able to reproduce most of the knowledge available on the early phases of the *F. tularensis* infection, but the authors claimed it could further provide insights into potential coadjutants of antibiotic therapies, aiming at stimulating macrophage activation. Finally, since it exceeds the scope of this review, we do not discuss here but want to mention the use of stochastic modeling in the simulation and prediction of epidemics spread of bacteria-associated lung infection diseases (Grundmann and Hellriegel, 2006; D'Agata et al., 2007; Agliari et al., 2013).

### Critical remarks on stochastic models

Stochastic models do not to scale well with the size of biochemical networks due to their structural complexity and the necessity to perform multiple realizations of the same simulation. However, the exponential increase in the computational power will make possible in the close future to simulate large stochastic models even in average scientific workstations. Calibration of stochastic models requires high sensitivity and specificity experimental techniques capable of quantifying random effects and fluctuations in molecule or cell abundance. For biochemical systems, this translates into single-cell technologies like single-cell transcriptomics, single-cell PCR, mass cytometry and fluorescence-based technologies (Crépieux et al., 1997; Lidke and Wilson, 2009; Spiller et al., 2010; Bakstad et al., 2012; Bendall and Nolan, 2012; Haack et al., 2013). Although these methods are to date technically challenging, expensive and not available in an average cell biology lab, one can foresee they will become standard technologies in relatively short time. Altogether, stochastic models are currently not suitable for systems that include many different interacting molecular or cellular species.

## Agent based models

### Main features of ABMs

**Figure 6:**
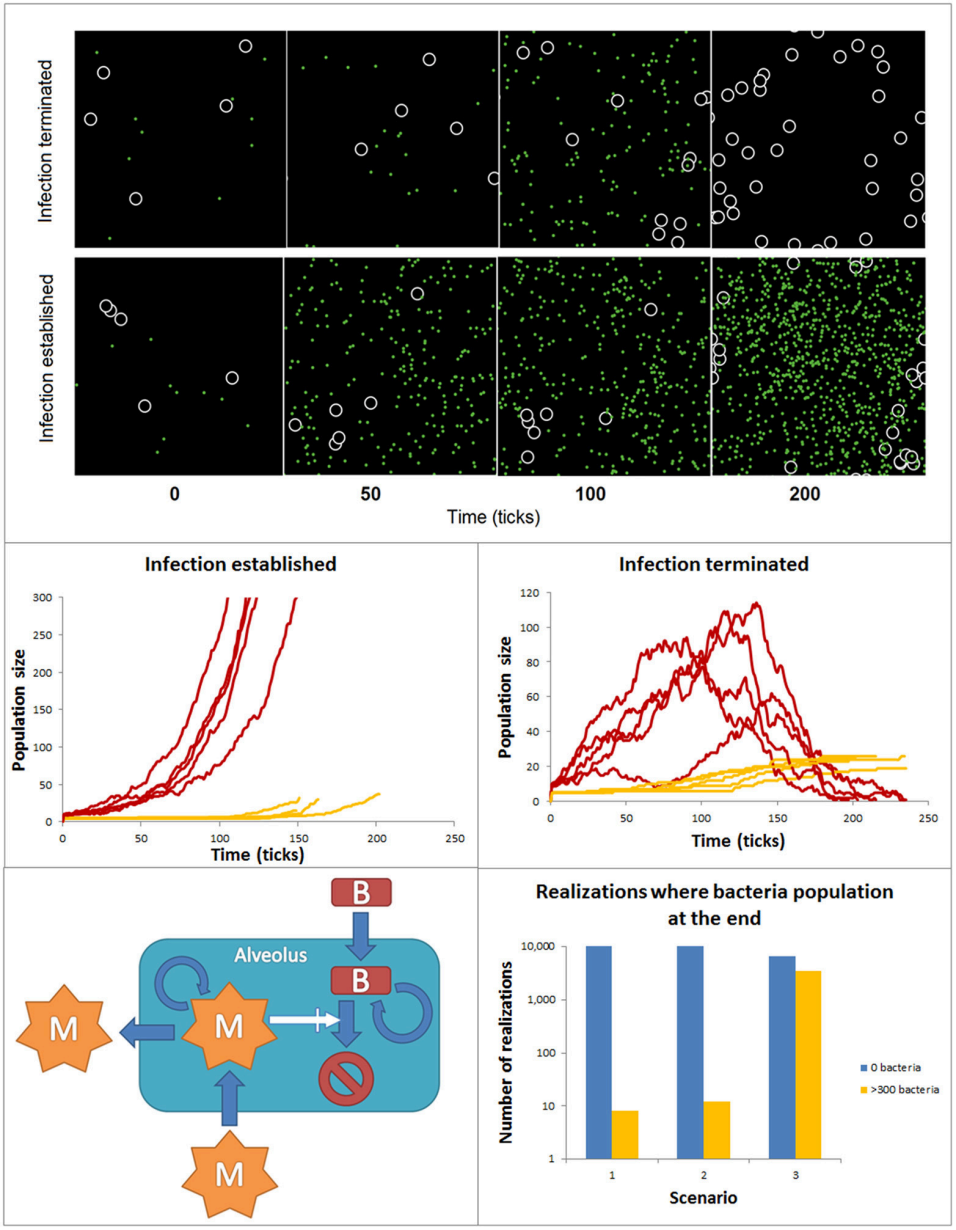
ABM accounting for the spatial features of bacteria and macrophage dynamics during early phases of lung alveolus infection. **Top:** Time course for bacteria and macrophage populations predicted by the ABM for simulations similarly initiated but reproducing infection resolution (top) and infection establishment (bottom). The black space represents an alveolus, white circles are macrophages and green dots are bacteria. Both populations can freely move within the alveolus. **Center:** 10 similarly initiated ABM simulations are classify into those accounting for infection successfully established (left) and those representing infection resolved (right). Red lines represent bacteria populations and yellow lines macrophage population. **Bottom:** ensembles of ABM simulations used to assess the relative importance of the processes modeled in the simulation output. 10^4^ simulations were implemented for each scenario. The solutions were divided into two groups (a) yellow bar: solutions ending in an establishment of bacterial infection [bacteria (t_i_) ≥ 300): and (b) blue bar: solutions ending with depletion of the bacteria population (bacteria (t_final_) = 0].

Many if not most of the intracellular biochemical reactions happen in complex, often highly crowded and heterogeneous spatial compartments (Rivas et al., 2004; Minton, 2006). Similarly, cell-to-cell interactions are affected by the features of the tissue compartments in which they take place. Logic networks, ODE or stochastic models have a relatively limited ability to account for spatial features. In contrast agent-based models (ABM) are powerful tools to simulate in a detailed manner the spatial features of these interactions at the single molecule or cell level. Agent-based models can be used to simulate the dynamics of ensembles of so-called agents in two and three dimensions predefined spaces. Agents are entities mimicking molecules or cells, which have the ability to simulate their movement within the modeled space compartment and their interactions with other species, also modeled like agents. The fate and movement of the agents depends on a set of rules, which are based on their molecular and cellular properties and the features of their interactions. ABMs can include a variety of different agent populations, which could operate at different spatial scales within the model. The **environment** surrounding the agents can display multiple spatial heterogeneous features, like spatial domains with different ability to diffuse or interact for the agents. Finally, the **rules** defining the update of the agent behavior can be the result of other models like ODEs or Boolean networks, but also stochastic rules. Ultimately, agent-based model simulations are intended to find collective, emergent patterns in the behavior of the agent populations. In the biomedical context ABMs have been primarily used to investigate interactions between cell populations. For example, in the early phases of infection both bacteria and macrophages are in low numbers and the spatial aspects of macrophage motility, sensing and recruitment, or bacteria motility and proliferation may decide the conditions for a fast resolution or a long- lasting extended infection. In these conditions, ABMs offer the possibility to simulate with detail the spatial features of the interaction between macrophages and bacteria in the lung alveolus. Figure 6 accounts for simulations made with an ABM. The ABM stands for the dynamics of two populations of agents accounting for bacteria and macrophages at the very early phases of bacterial lung infection. Thus, the infection is assumed to take place in a single alveolus and both agents are assumed in low numbers when the simulations are initiated. The alveolus is modeled like a torus shaped surface of 32 × 32 pixels. The macrophages are 2 pixels wide and bacteria are considered to be non-dimensional dots. During the simulations, bacteria and macrophages move in 1 pixel. In the simulations, the time is discrete, with time iterations in the time-scale of the processes considered. As initial conditions for the simulations, the initial amount of bacteria and macrophages are situated in random positions of the 2D space. The behavior of each individual agent is governed by a set of rules describing the ability of macrophages and bacteria to move, the bacteria proliferation, the recruitment of monocyte-derived macrophages and the bacteria killing after bacteria-macrophage encounter (See Supplementary Material for more details). To make the model more accurate, we assumed the stochasticity for the bacteria movement and proliferation, as well as for the macrophage movement and recruitment. Thus, the evolution and final fate of two similar simulations can differ drastically. For example, Figure 6 top displays the time course for bacteria and macrophage populations during two similarly initiated simulations with 250 time units duration, which display totally different time courses. In the top simulation, the bacteria infection is resolved and the bacteria population gets extinct, while the bottom simulation ended with a successful bacterial colonization although the initial conditions were very similar.

In many ABMs like in this one, a number of the processes models are described by stochastic rules. Thus, the simulations become stochastic and to detect patterns of regulation ensembles of ABM simulations are analyzed using statistical methods. In our example, we performed a series of simulations and classify them in two groups of 5 simulations (Figure 6 center): (a) those in which at the end of the simulation the population of bacteria is extinguished and (b) those in which the population of bacteria reaches 300 individual in the course of the simulation, used as indicator that the bacteria colonization has been established and the infection has extended to surrounding alveoli. In line with this, ensembles of predictive simulations can be used to assess the relative importance of the processes modeled in the simulation output. For example, we used the model to assess the effect on the success of bacteria colonization of higher proliferation rate of bacteria invasion (scenario 2) and decreasing infiltration of macrophage (scenario 3, Figure 6 bottom). Scenario 1 defines the control situation. To make this analysis, we run 10^4^ ABM simulations for each scenario, and counted the number of simulations per scenario in which the bacteria population was extinguished (blue bar) or the bacteria colonization was successful (orange bar). The results show a certain level of stochasticity and suggest that decreased efficiency in monocyte-derived macrophage recruitment has more impact in fostering bacteria colonization than increased bacterial proliferation rate.

### Examples of ABMs in literature

Chavali et al. made a detailed discussion of the use of ABMs to investigate and characterize emergent properties of immunological systems (Chavali et al., 2008). ABMs have been used to model in detail the spatial features of molecular interactions within cellular compartments, for example, the dynamics of molecules in cell membranes (Haack et al., 2013; Santos et al., 2016). In line with this, Rhodes et al employed agent-based modeling to analyse the spatial features of the cytoplasmic dynamics for the NF-κB inhibitor IκBα (Rhodes et al., 2015). It has been found that IκBα can co-localize and get sequestered in cytoskeleton structures like the microtubule organizing center and the α-tubulin filaments (Crépieux et al., 1997). To model in detail this process, Rhodes and co-workers derived a model for the NF-κB activation via type 1 IL-1 receptor (IL-1R1). The model considers: (1) activation of NF-κB through IL1R; (2) activation of anti-apoptotic pathways via PI3k signaling; and (3) cytoskeleton reorganization during the NF-κB activation through Ras activation. Using model simulations, the authors hypothesized that the sequestration of IκBα can be a mechanism to modulate the intensity of the L1RI input signal coming from L1RI when transduced inside the cell. The mobilization and/or sequestering of signaling proteins to microtubules and other cytoskeleton structures has been found in other key pathways for inflammation like MAPK cascades (Hanson et al., 2007), which indicates that the use of ABMs to dissect the fine-tuning of this mechanism may render interesting mechanistic hypothesis.

ABMS can also be used to establish the link between molecular interactions and cell phenotypes. In line with this, Stern et al. used an ABM to simulate the response to damaged tissue and barrier disruption signals of individual epithelial cells embedded in an extracellular matrix (Stern et al., 2012). In many infectious diseases including pneumonia, the breakdown of the epithelial barrier exposes the inner part of the organism to external pathogens and facilitates their systemic spread and the emergence of sepsis. In the model used, the agents account for the epithelial cells and the rules for the effect on them of the activation of the EGF and TGF-β receptor mediated signaling pathways. It has been found that down-regulation of TNF-α signaling and activation of EGFR signaling contribute to the maintenance of epithelial barrier integrity and function in lung and other epithelial tissues (Finigan et al., 2012; Patel et al., 2013; Uwada et al., 2017). The model was able to simulate tissue damage and wound recovery. Moreover, the model simulations suggested the existence of a mechanism for the crosstalk between TGF-β and EGFR pathways involved in the recovery after damage. The activation of these pathways have been linked to the response alveolar epithelial cells to some types of bacterial infection (Choi et al., 2011; Li et al., 2015).

ABMS can also be utilized to dissect the spatial features of cell-to-cell interactions in their natural tissue compartments. In order to investigate T cell (TC) activation Bogle and Dunbar built an ABM (Bogle and Dunbar, 2010). The model attempted to investigate the spatial features of TC activation by active dendritic cells (DCs) in the lymph node, thereby trying to establish mechanistic links between the properties of TC and DC motility in the lymph node and the timing and strength of the TC response elicited. The processes included in the ABM were the proliferation of TCs in lymph nodes, the DC driven activation of lymphocytes, and the DC and TC trafficking through the lymph node. The model was used to simulate the proliferation, release and changes in the affinity profile of TCs in the lymph node. The simulation results correlate with data accounting for the efflux rate of activated TCs from lymph nodes. Further, model analysis and simulation were used by the authors to point to open questions and gaps in the current knowledge of the TC-DC interaction in lymph nodes. For example, they hypothesized that the deeper understanding of TC activation can benefit from experiments elucidating the dynamics of the lymph node vascularization, a process that seems to be modulated by the DCs (Webster et al., 2006).

Moreover, ABMs can be used to study in detail spatial properties of infection-related autocrine and paracrine loops. In a work on chronic asthma, a condition we already linked to lung infection, Pothen et al. (2015) hypothesized that in healthy individuals antigenic stimulation drives both the onset and the recovery after allergic inflammation. Under these conditions, allergic inflammation can become a self-limited event. Based on this idea, Pothen et al. used modeling to investigate under which conditions a failure in this process can provoke the chronic airway inflammation associated to asthma. To this end, they derived an ABM that considers spatial features of the interactions between pro- and anti-inflammatory cells during tissue damage and repair in unresolved allergic inflammation. Models simulations suggested that the ability to recover after the allergic episode is in general terms very robust regarding most of the pro- and anti-inflammatory cells interactions, but appears very sensitive to increase in the recruitment and activation of pro-inflammatory cells like neutrophils and eosinophils. The model simulations indicated that down-modulation of pro-inflammatory cell activation could be a therapeutic strategy against the allergic inflammation.

ABMs can be used to mimic the effect of cell exposure to diffused extracellular ligands, biomolecules and non-organic particles. Brown et al. used an ABM to investigate lung inflammation and fibrosis following particulate exposure (Brown et al., 2011), an environmental condition that can increase the chances and severity of lung infection (Mehta et al., 2013). The model accounted for the interaction between lung macrophages and fibroblasts through TNFα and TNFβ. It also considered the tissue damage caused by TNFα and the production of collagen for repairing the tissue. The model simulations predicted three main states for particulate exposure associated lung inflammation: (1) self-resolving inflammation, (2) localized tissue damage and fibrosis and (3) elevated pro and anti-inflammatory cytokines and persistent damage. Model simulations showed that the switch between the different states depends on the intensity and duration of the exposure to the particulate damage.

### Critical remarks on ABMs

ABMs can deal with systems that are complex and heterogeneous from a spatial perspective, but also with biological systems involving many different interacting entities, cell and/or molecules, and multi-levels. The essentially modular structure of ABMs facilitates the addition of new types of agents, accounting for new cellular or molecular players. Even simple rules defining the interactions between the agents can generate extremely complex spatio-temporal regulatory patterns. However, to date these models do not scale well with respect to the number of total interacting agents due to the large computational resources necessary to simulate systems with large number of agents. In line with this, a lot of work has been done in the last decade in terms of methods for efficient and distributed ABM simulation (Aaby et al., 2010). Further ABMs are suited for performing detailed simulations, but very poor in terms of analytical tools. Far from the much elaborated algorithms conceived for the calibration of ODE and PDE models, very little has been done in terms of the systematic integration of quantitative data into ABMs (Bianchi et al., 2007) and computational tools specially designed for modeling of biological systems (Kang et al., 2014; Starruß et al., 2014). In any case, we think that ABMs will be an interesting alternative in the coming future for modeling bacterial lung infection.

## Discussion

### Great expectations for mathematical modeling in lung infection and inflammation?

We have great expectations in terms of what mathematical modeling can contribute in the coming decade to the understanding of lung infection pathophysiology. In the last years modeling has been used in biomedicine essentially for integrating multiple types of experimental data, formulating mechanistic hypotheses, or in performing simulation-based therapy assessment. However, mathematical modeling can be used in many other avenues that are not yet sufficiently tested in pulmonology. Epstein (2008) suggested up to 16 motivations other than pure prediction to use modeling and simulation in science. In Table 1 we have selected a few of them and elaborate how they could be implemented in the context of bacterial lung infection.

**Table 1:** Ten “not-yet-considered” motivations to use mathematical modeling in bacterial lung infections.

**Motivation**	**Usage in bacterial lung infection**	**Article of example**
Discover new questions	Combination of model derivation, simulation and analysis used to formulate in a consistent manner a new hypothesis on the early steps of bacteria lung infection mechanism	Gillard et al., 2014
Guide data collection	Model simulations used to (a) decide the design of the which most suited experiments to test the above hypothesis and (b) select relevant public available data	Thakar et al., 2007
Explain (very distinct from predict)	Customized model simulations used to (a) illustrate the experimental results and (b) discuss/extrapolate the consequences of the proved or disproved hypothesis	Smith et al., 2011
Illuminate core dynamics	A model comprising the core of the network controlling inflammation used to point the key molecules and processes controlling it	Krishna et al., 2006
Reveal the apparently simple to be complex	The analysis of a model representing the apparently simple and small network controlling early bacterial lung infection used to suggest the existence of non-linear behavior associated to feedback loops circuits	Nikolov et al., 2010
Reveal the apparently complex to be simple	Model reductions techniques on a large network representing bacterial lung infection applied to detect the few key processes and molecules controlling the process	Guo et al., 2011
Expose prevailing wisdom as incompatible with available data	Simulations of a mathematical model encoding the current knowledge on molecular interactions controlling initiation of inflammation employed to show inconsistencies with new data	Hoffmann et al., 2002
Bound outcomes to plausible ranges	Comparison between model simulations and available data used to establish the interval of biologically feasible features (parameters) for bacteria proliferation and spread in the lung alveoli	Mochan et al., 2014
Offer crisis options in near-real time	For a patient entering the Intensive Care, personalized model simulations used to predict the course of the host-pathogen interactions and near-real time decide on the therapeutic alternatives	Dix et al., 2016

To mention an interesting open question, some immune cell types have a dual, often ambiguous role during infection. For example, macrophages and neutrophils are major players in the quick resolution of infection, but under exacerbation they can also worsen the condition by promoting tissue destruction or overwhelming inflammation (Nouailles et al., 2014). This duality can be explained at least in part by the deregulation of intra- and inter-cellular positive feedback loops working often in an autocrine or paracrine manner. For example, TNFα can be secreted by activated macrophages to signal other immune cells in early lung infection (Mukhopadhyay et al., 2006), but it can at the same time promote activation of resident or monocyte-derived macrophages in a amplification loop that can exacerbate local inflammation (Gane et al., 2016). The use on mathematical models dissecting the structure and fine regulation of these circuits can contribute to the understanding of this aspect of acute lung infection.

Moreover, a number of infections and inflammatory conditions in the lung like asthma and tuberculosis persist despite treatment and reappear in an episodic or cyclic fashion. This suggests that autocrine and paracrine regulatory circuits, including positive and negative feedback loops may get disrupted and deregulated in the course of these diseases. For example, G-protein-coupled adenosine receptors have been associated to protection from tissue damage in infection and sepsis (Csóka et al., 2010). Further, adenosine has been linked to the pathogenesis of asthma (Brown et al., 2008). This role is mediated via a physiological negative-feedback mechanism that seems to participate in limiting and terminating tissue-specific and systemic inflammatory responses (Ohta and Sitkovsky, 2001). Mechanistic mathematical modeling of this type of paracrine feedback circuits may shed light into their role controlling overwhelming immune response and the consequences of their deregulation.

Modeling has been used for longtime in pharmacology to assess the efficacy and dosage of drugs. Moreover, model simulations in combination with computational sensitivity analysis and model optimization have been used to detect new potential drug targets in cancer and metabolic diseases, or to assess the emergence of therapy resistance (Vera et al., 2007; Schoeberl et al., 2009). This strategy can be replicated in lung infection diseases to search for new drug targets or repurpose existing drugs as immunomodulators during lung infection (Wentker et al., 2017), or to optimize the current protocols for antibiotics administration (Schirm et al., 2016). Further, in recent times (Zhou et al., 2017) modeling has been used to assess therapies in a personalized manner (Rosenberg and Restifo, 2015; van de Sant et al., 2017), especially anticancer ones (Gupta et al., 2016). We think there is potential for this in lung infection and pneumonia, by integrating selected patient unique –omics and physiological parameters into model simulations, and use them to customize treatments.

### Mathematical modeling and multi-level dissection of bacterial lung infection: the art of choosing the right approach

There is no perfect modeling framework for investigating bacterial (lung) infection in all possible scenarios. This is because the optimality of a modeling strategy will depend on the aim of investigation, the scale and structural complexity of the system to be modeled and the quantity, quality and nature of the experimental data available for its characterization. Table 2 extends our previously published table (Vera and Wolkenhauer, 2011) and compares the main modeling frameworks here discussed based on a number of important features. We also include some prototypical case studies in bacterial lung infection in which each modeling framework could be most suited. One can see that there is no a modeling approach clearly superior to all the others for every feature analyzed, and therefore the choice of the right model relies often on a tight balance between several of these features (Table 3). Moreover, in some cases any of the methodologies described displays the features necessary to model the dynamics of given biological systems, and other modeling frameworks can be used (See Supplementary Material for further discussion).

**Table 2:** Features of different model formalisms analyzed.

**Modeling framework**	**Realism**	**Time**	**Scalability**	**Computational cost**	**Complexity**	**Data usage**	**Examples**
ABM	Phenomenological	Continuous	Large	High	High	Low	Spatial simulation of cell-2-cell interaction and movement in a lung alveolus during infection
Boolean	Phenomenological	Discrete	Medium	Low	Low	Medium	Analysis and simulation of the large regulatory network triggered in macrophages after bacteria detection
ODE	Mechanistic	Continuous	Small	High	Medium	High	Analysis of fine-tuning of NFkB signaling activation in lung epithelial cells after infection
Stochastic	Mechanistic	Continuous	Small	High	High	High	Simulation of dynamics of few bacteria initiating infection in a lung alveolus
Fuzzy Logic	Phenomenological	Discrete	Medium	Medium	Low	Medium	Simulation of lung epithelial cell phenotypes with uncertain uncomplete information of activators

*Realism: How close from the real biological mechanism is the representation given by the model; time: Whether the model handle the time as a discrete or continuous variable; scalability: Number of compounds the model can on average handle (small: up to 20 compounds, medium: 20–100, large 100–1,000; computational cost: Time and computational resources demanded for model simulation and analysis; complexity of the models in terms of their structure; data usage: Whether the construction of the model requires low, medium or large amounts of quantitative experimental data for its characterization; examples: Possible applications for each formalism in the context of bacterial lung infection*.

**Table 3:** Applicability of different model formalisms analyzed into different biological situations.

**Modeling framework**	**Intracellular circuits**	**Cell–cell interactions**	**Host-pathogen interactions**	**Local tissue interaction**	**Systemic infection/inflammation**
ABM					
Boolean					
ODE					
Stochastic					
Fuzzy Logic					

*Applicability: This table presents an illustrative guidance to select the best modeling framework to the biological scale of interest. Depending on the scale the applicability of the different frameworks can be poor (black) possible (gray) or appropriate (white)*.

**Figure 7:**
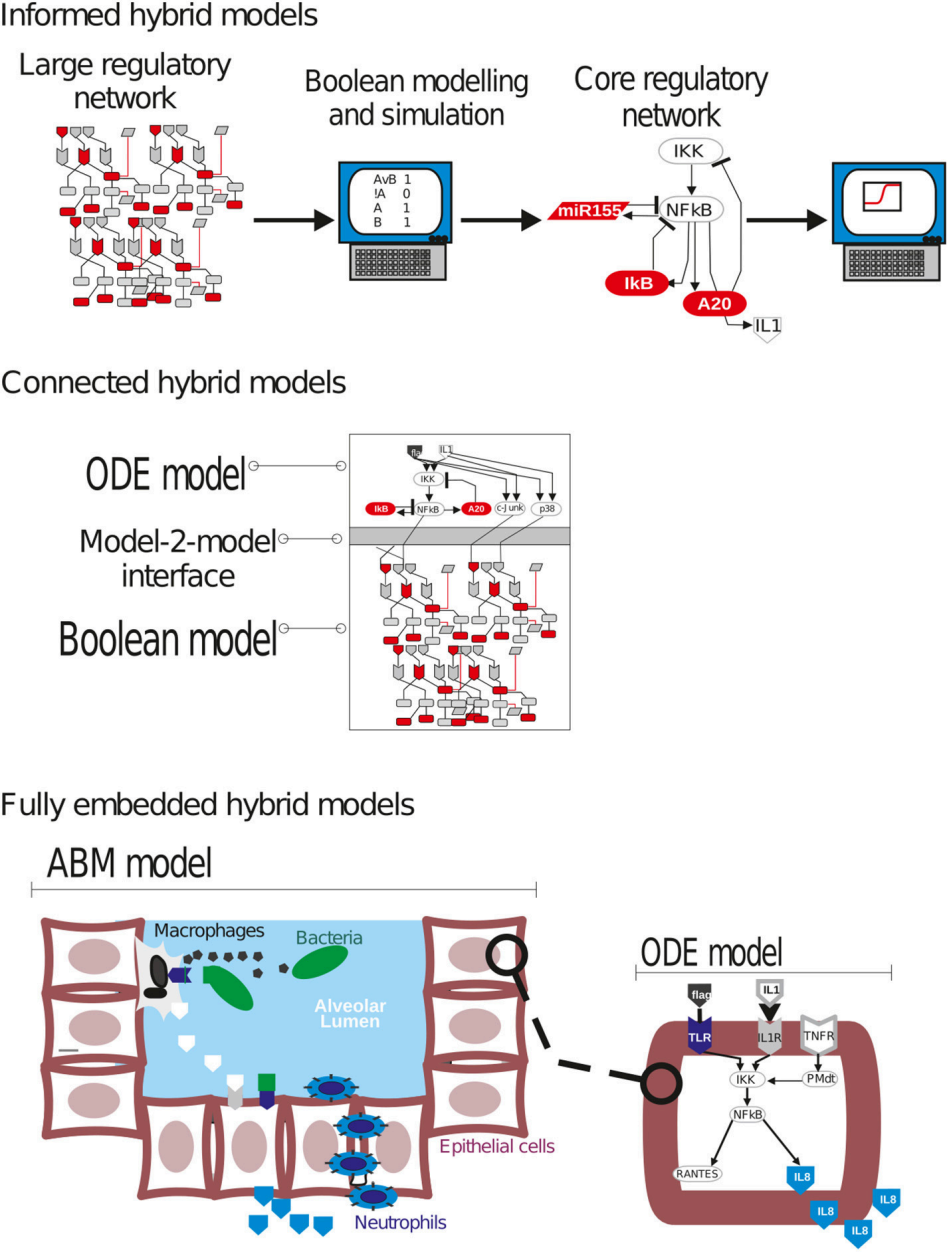
Different strategies for hybrid modeling in bacterial lung infection.

In some cases a single modeling approach is not sufficient to deal with some structurally complex systems, and one has to combine different model types into a “hybrid model” (Chiam et al., 2006; Wylie et al., 2006; Wu and Voit, 2009). Agent-based models has become the most used approach in biomedicine for multi-level and multi-scale systems (Chavali et al., 2008). However, other hybrid modeling strategies are implemented by combining modeling approaches with computational and knowledge requirements of different complexities, like Boolean and ODE model together (Figure 7). For example, one can use the knowledge generated by simulations with a given type of model to parameterize and characterize a second type of model. In this “**informed hybrid models**” there is no formal connection between the models, but one of them is used to design or characterize a second one. For example, In Rex and collaborators simulations on a large Boolean network were used to describe the key regulatory circuits underlying the shift between M1 (classical, LPS-activated, pro inflammatory) M2 (IL4/IL13 activated, anti-inflammatory) macrophage phenotypes (Rex et al., 2016). This information, the key molecular species and their interactions, was used to construct a second ODE model that dissects the fine regulation of this subnetwork.

Another option could be to construct models in different frameworks that are primarily independent, but cross-talk via a few common compounds. An example of this “**connected hybrid models**” could be a combination of an ODE model accounting for a signaling circuit controlling the activation of a number of key transcription factors after bacterial infection (e.g., NF-κB, p38), connected to a large Boolean network accounting for the activation of dozens to hundreds of transcriptional targets. The connection between both types of models could be done via interface functions accounting for the activation status of the transcription factors (Khan et al., 2014).

Finally in the “**fully embedded hybrid models**” a model in a given formalism is fully integrated in another type of model (Chiam et al., 2006). We think this is an alternative in which ABM could be a suitable option. For example, in multi-scale models accounting for bacterial lung infection one could develop an ABM in which individual bacteria, lung epithelial cells, alveolar macrophages or neutrophils populations are modeled like interacting agents moving within a defined space. The activation, differentiation or apoptotic phenotypes of these agent-cells would be determined by the simulation of embedded Boolean or ODE models, which describe the time-dependent activation of their core intracellular network.

## Author contributions

All authors listed have made a substantial, direct and intellectual contribution to the work, and approved it for publication. MC and PW wrote the section of the boolean section, GS wrote the section of ODE and ABM, JV wrote the introduction and stochastic models section. XL provided a critical review on the whole manuscript. All authors wrote the discussion and made the final correction to the article.

### Conflict of interest statement

The authors declare that the research was conducted in the absence of any commercial or financial relationships that could be construed as a potential conflict of interest.

## Glossary

**Agents**: The elements in an ABM that interact in the environment.
**Bifurcation analysis**: Follow the changes in the qualitative behavior of the simulation of a kinetic model after modifying one parameter.
**Bistability**: A property that kinetic models can present. It is characterized by two stable steady solutions of the system.
**Design space analysis**: Identify regions defined by a couple of parameters that show different qualitative behaviors depending on the values of these parameters.
**Digital response**: Response to stimuli by activating key molecules and phenotypes in an all-or-nothing manner.
**Electronic Logic Gates**: Elementary electronic block of an ideal circuit that performs logic operations. Usually, each block is characterized by two inputs and one output; logic operations that are performed are: AND, OR, XOR, NAND, XNOR, and NOR. The logic operation with one input and one output line is NOT.
**Environment**: The open area in which is defined an ABM.
**Kinetic model**: Mathematical model which consider the time dimension in the simulations.
**Mass-Action**: Mathematical formalism that represents the velocity of the processes as the product of the elements interacting rose to integer values. These kinetic orders correspond with the stoichiometric values of the interaction.
**Model calibration**: Searching for the values of the parameters that produce a simulation from a kinetic model that reproduce the dynamic behavior of experimental data.
**Model optimization**: Searching for the parameter set that best reproduce a specific simulation of interest by a kinetic model.
**Parameter identifiability**: Problems of certain systems to find a precise value for the parameters given a set of experimental data. This problem ends with a broad uncertainty on the values of some parameters.
**Rules**: The definition of the possible interaction that the elements can have.
**Sensitivity analysis**: A mathematical analysis to quantify the effect of the parameters of the model on the response of the simulations.
**Symbolic analysis**: A qualitative analysis of a kinetic model allowing to identity general patterns without giving specific values to the parameters of the model.
**Synchronous and asynchronous algorithms**: These methods refer to the update of the nodes' states. In the first case, all nodes are updated during each iteration step following the set of functions defined in the model. Asynchronous updated introduce uncertainties typical of biology: during one iteration, only some nodes randomly chosen are updated accordingly to the defined set of functions.
**T cells**: A branch of the immune response key for the mid-term response to infection.
