# Corrigendum: Multiplicity of Mathematical Modeling Strategies to Search for Molecular and Cellular Insights into Bacteria Lung Infection

**DOI:** 10.3389/fphys.2017.00817

**Published:** 2017-10-30

**Authors:** Martina Cantone, Guido Santos, Pia Wentker, Xin Lai, Julio Vera

**Affiliations:** Laboratory of Systems Tumor Immunology, Department of Dermatology, Friedrich-Alexander University Erlangen-Nürnberg and Universitätsklinikum Erlangen, Erlangen, Germany

**Keywords:** systems biology, systems medicine, lung infection, mathematical modeling, Boolean network, ODE models, stochastic modeling, agent-based modeling

In the original article, there was a mistake in the legend for Figure 5 as published. In the legend the red color should represent bacteria and the blue color macrophages. The correct legend appears below.


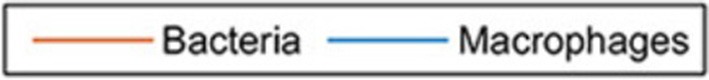


In the original article, there was a mistake in the legend for Table 3 as published. In the caption the legend for the black color should refers to “appropriate” and the white color should refers to “poor.” The correct caption appears below.

Applicability: This table presents an illustrative guidance to select the best modeling framework to the biological scale of interest. Depending on the scale the applicability of the different frameworks can be poor (white) possible (gray) or appropriate (black).

In the original article, Kholodenko (2006) was not cited in the article. The citation has now been inserted in Introduction, fourth paragraph and should read:

A balanced immune response can be achieved via interacting immune cells that are controlled by intracellular regulatory networks of interacting molecules, such as cytokines, receptors, kinases, transcription factors, or non-coding RNAs. Such a system contains regulatory motifs, especially positive and negative feedback loops, which increase the complexity of the response and can provoke non-linear behaviors such as bistability and oscillation (Kholodenko, 2006). For patients with respiratory bacterial infections, severe pathological condition can emerge if their immune systems fail to quickly neutralize the infection and to avoid systemic spread of the pathogen. On the other hand, overwhelming host immune response to the pathogens is also dangerous and can impede the proper functioning of the lung and other organs. So, any new treatments using the combination of antibiotics and immunomodulatory drugs will be useful if they can help the patients to maintain a balanced immune response (Wentker et al., 2017), which is governed by the multi-level biological system (Eberhardt et al., 2016).

## Conflict of interest statement

The authors declare that the research was conducted in the absence of any commercial or financial relationships that could be construed as a potential conflict of interest.
