# Supplementary material for: Multiplicity of Mathematical Modeling Strategies to Search for Molecular and Cellular Insights into Bacteria Lung Infection
Source: Front Physiol. 2017 Aug 30;8:645. doi: 10.3389/fphys.2017.00645 (PMC5582318; doi:10.3389/fphys.2017.00645)
Supplement: Supplementary file 1 [file Table1.DOCX]

**Multiplicity of mathematical modelling strategies to search for molecular and cellular insights into bacteria lung infection**

**Supplementary Material**

Martina Cantone^*^, Guido Santos^*^, Pia Wentker, Xin Lai, and Julio Vera

Laboratory of Systems Tumor Immunology, Department of Dermatology, Friedrich-Alexander University Erlangen-Nürnberg and Universitätsklinikum Erlangen, Erlangen, Germany

^*^ Equal contributors

Corresponding author: Prof. Dr. Julio Vera, Phone: +49 9131 85 45876, Fax: +49 9131 85 33701, E-mail: julio.vera-gonzalez@uk-erlangen.de

**Other mathematical modelling approaches**

The modelling approaches discussed above represent a selection of the most used formalisms in biomedicine. However, they are not the only ones that have been or can be used. In the following, we briefly mentioned and discussed other modelling methodologies that have been applied to date.

Bayesian probability has become very popular in many fields as it permits to incorporate *a priori* knowledge about the biological processes investigated. One way to consider the previous knowledge into a mathematical model is using Bayesian Networks(1). This approach is similar to the Boolean modelling in terms of is graphical representation of the models as networks of interactions between elements (molecules or cells), but here the model variables are random and the interactions are represented as conditional dependencies(2). In Nemzek and coworkers(3) a Bayesian networks was used to analyse data describing direct or indirect damages of lung tissue with different levels of severities. Bayesian analysis concluded that both the level of severity in lung injury and the presence of sepsis affect the strength of neutrophil recruitment.

One approach including specifically the space dimension discussed so far is agent-based modelling. However agent-based models make a coarse–grained description of the spatial features. When a more mechanistic description of the system’s spatial scale is needed, an appropriate approach can be the modelling in partial differential equations. Here, the models consist of a set of differential equations in which the derivatives are also evaluated in the space dimension. To make the models computable, the space dimension is discretized and the space movement of the species is modelled using flow equations through the discretized space. Consequently, the number of variables and the complexity of the model is comparatively much higher than in an ODE model, and so the complexity and computational cost of this approach. For example, the Virtual Cell provides a general computational framework for modeling at this scale the chemical and electrophysiological processes in living cells(4). In the context of lung infection, Friedman and coworkers derived a partial differential equations model to dissect the behavior of granulomas, a small tissue-like structure containing immune cells that wall off Mycobacterium tuberculosis pathogens, the causative agent of tuberculosis(5).

An entire family of computer-oriented models, often written in high-level programming languages and conceived for being built, run and executed directly in computing machines have also been used for modelling in biomedicine in the last decade(6). A paradigm of these models is Petri nets, a class of directed bipartite graphs. They contain two types of nodes: transitions, represented by bars and accounting for molecular activities; and places, drawn like circles and accounting for molecules. The directed arcs are represented by *arrows*, connect the nodes and indicate which places (that is, molecules) are pre-conditions (input or substrate) or post-conditions (output or product) for each of the transitions. Since Petri nets were designed *ex professo* for the utilization in computers, they benefit from computational features like scalability, modularity, and efficient execution and visualization. For an introduction to Petri nets in biochemical systems the reader is referred to Heiner et al. 2012(7). Interestingly, a few recent examples illustrate the use of Petri nets in immunology(8). Peng et al. employed Petri Nets modelling to make a comprehensive reconstruction of the NF-κB signaling network(6). Carvalho et al. derived and characterized a multi-level Petri net model to represent the mycobacterial infection (the pathogen responsible for tuberculosis) and the subsequent innate immune response10).

Other than Boolean logic, there are modelling approaches are able to deal with genome-scale networks, but in general terms they are suited only for metabolic networks(8,9). In Ferrarini et al.(10) a genome-scale metabolic model was reconstructed for three pathogenic mycoplasma bacteria species. The models, which were calibrated using nuclear magnetic resonance data, were able to explain the difference in pathogenicity between different mycoplasma species.

**Case Study: core pathway controlling inflammation in lung epithelial cells.** Inflammation signaling is a key process during the early phases of bacterial infection in which resident macrophages and epithelial cells trigger the immune response against the invading pathogens. In essence, upon the interaction of conserved bacteria antigens with plasma membrane receptors the inflammatory signaling cascade is activated in the host cell. This leads to the activation of intracellular gene programs promoting the secretion of ligands such as interleukins that mediate and amplify the activation of a first local and later systemic immune response. The core pathway regulating the inflammatory response is the NF-κB pathway. Here, we modelled the central part of the NF-κB pathway by extending preliminary mathematical modelling efforts(11–13).


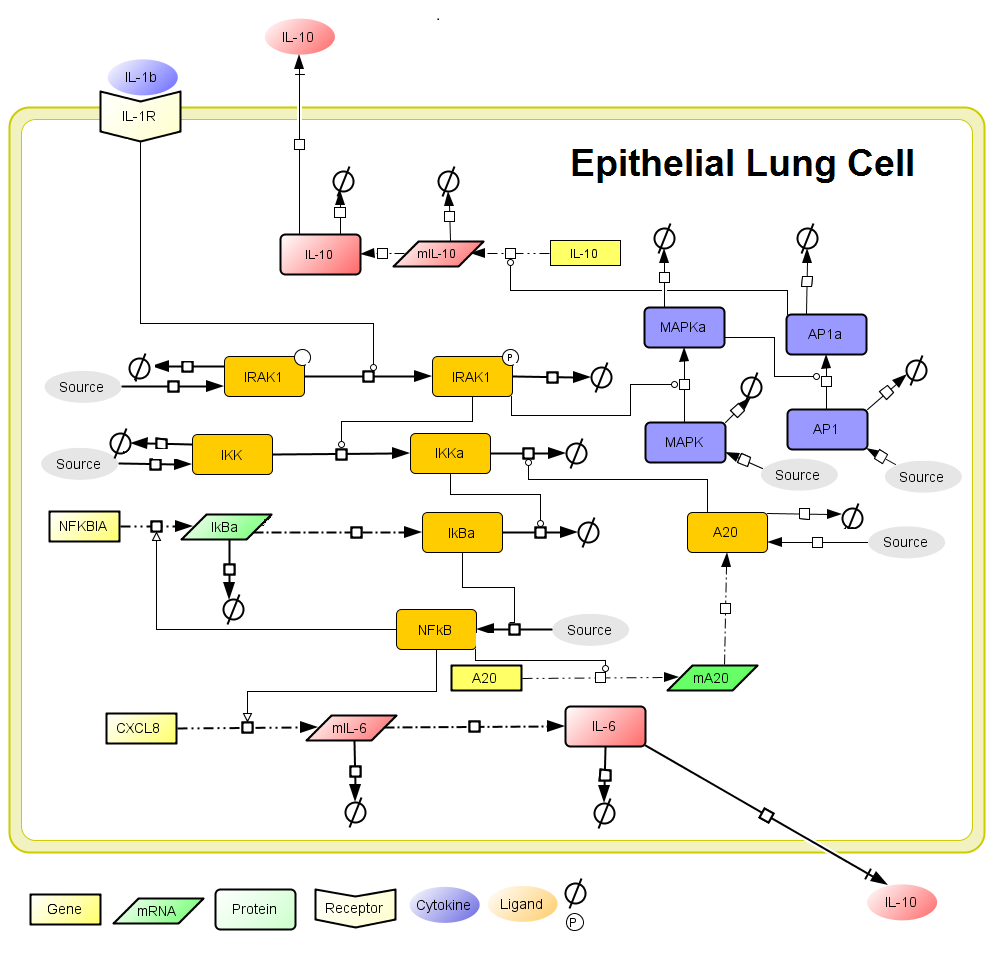


Figure 1. *CellDesigner* representation of NF-κB signaling pathway modeled with ODE model.

In the pathway (Figure 1), the interleukin IL-1β triggers the activation of the corresponding receptor provoking the phosphorylation and activation of the intracellular protein mediator IRAK1, which in turn activates IKK. Active IKK induces the activation and subsequent nuclear translocation of the transcription factor NF-κB by phosphorylation and further degradation of its inhibitor IκBα. Once in the nucleus, NF-κB triggers a complex transcriptional program that includes the secretion of several interleukins like IL-6, but also the transcription of IκBα and A20 in a sort of sophisticated negative feedback loop conceived to tightly control the timing of NF-κB activation(11,13,14). In our model, the activation of the MAPK cascade promotes the transcription of IL-10 upon activation of IRAK1. This pathway consists of a sequence of activation phosphorylation reactions. Active p38 promotes the nuclear localization of the transcription factor AP1a, which in turn activates the transcription and subsequent secretion of IL-10(15). The ODE model is a system of 15 differential equations.

Simulation of the model

We here used the mathematical model to analyze the effect that have deactivating mutations in key genes of the NF-κB pathway in the net secretion of interleukins by lung epithelial cells. Top panel of Figure 2 is a predictive simulation accounting for the production of IL-6 and IL-10 in response of IL-1β mediated NF-κB activation in different conditions. The wild type condition is displayed in blue. In addition we showed the predicted time profile for both cytokines under deactivating mutations of IRAK1 (here represented as IRAK1^-^ and IKK (IKK^-^). In order to simulate the mentioned mutations, a) the value of the rate constant accounting for the activation of IRAK1 is decreased 90% with respect to the nominal value (the nominal value solution was arbitrarily defined in a way it resulted biologically feasible and instructive for the purpose of this review) and b) the rate constant accounting for the IKK-mediated NF-κB nuclear import is decreased 90%. The model predictions indicate that the IRAK1 mutation (IRAK1^-^) has a significant effect in the production of IL-10 and can lead to a 50% decrease in its maximal concentration. Similarly, IKK mutation (IKK^-^) reduces the secretion of IL-6 while not affecting IL-10. Interestingly, the predictive simulations are confirmed by published experiment reports, as suggested by Table 1.

| 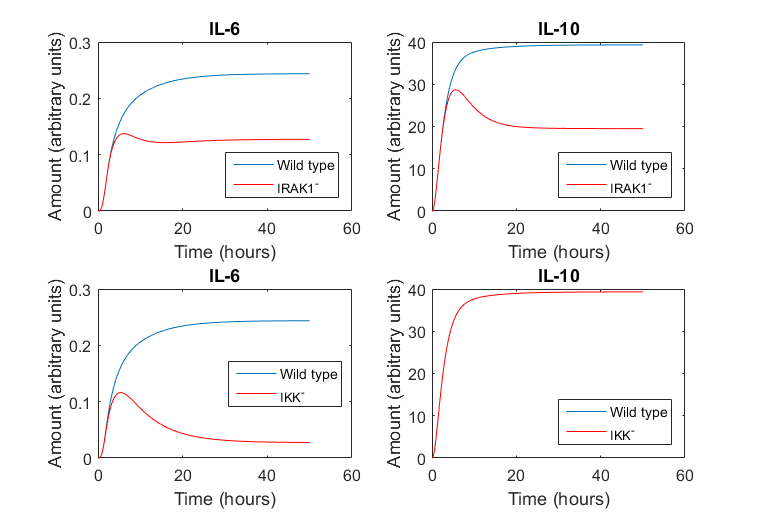 A  B  C  D | |
| --- | --- |
| 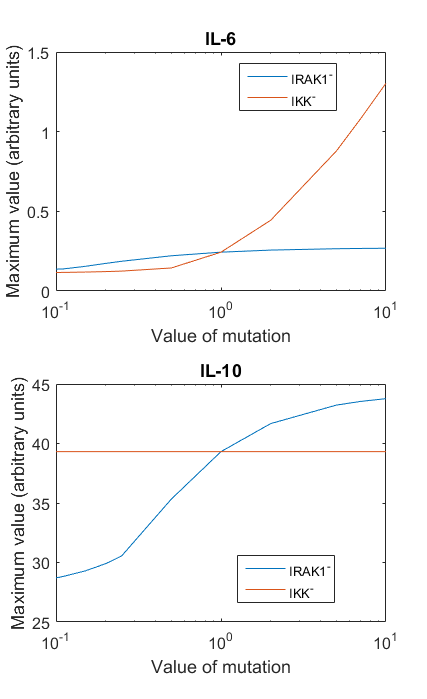 | 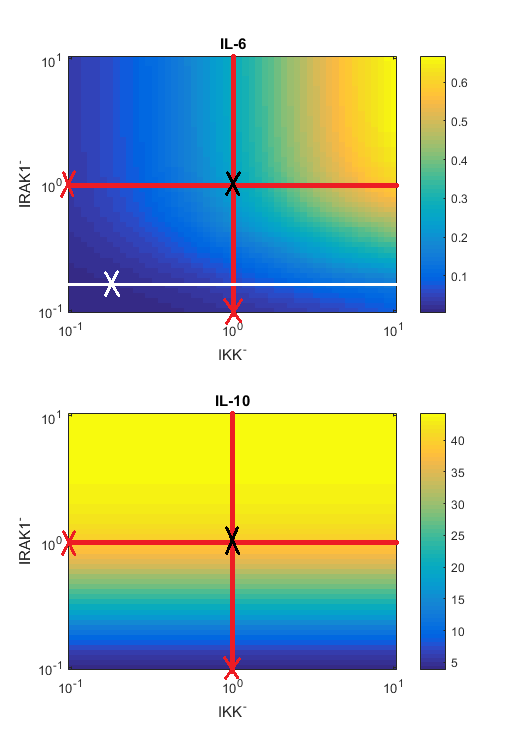 |

Figure 2. Top panel: model simulation of the NF-κB signaling pathway by ODE formalism. A, B, Comparison between wild type simulation (blue line) and IRAK^-^ mutation on the system (red line). C, D, Comparison between wild type simulation (blue line) and IKK^-^ mutation on the system (red line) (IL-10 curves overlapped in panel D). Bottom left: effect of systematic variation on the value of the mutation, values of mutation higher than 1 means increase in the velocity of the process, values lower than 1 means decrease in the velocity of the process. The value of the curves represents the maximum of the simulation for IL-6 and IL-10 after the mutation. Bottom panel on the right: region of different combination of mutation in IKK and IRAK1and their effect on the maximum value of IL-6 and IL-10. Black cross indicates the wild type condition. Red lines represent the path of single mutations indicated in panel bottom left of Figure 6 of main text. Red crosses indicate the single mutations represented in panel top right. White cross indicate an arbitrary mutation that makes IL-6 production independent to mutations in IRAK1 (through white line).

|  | IRAK^-^ | IKK^-^ |
| --- | --- | --- |
| IL6 | Decreased^1^ | Decreased in some patients^2,3^ |
| IL10 | Decreased^4^ | No effect^2^ |

Table 1 : Experimental results(16–19). Effect on IL6 and IL10 production after mutation of certain components of the NF-κB signaling

We further performed systematic predictive simulations, by changing the values of the indicated kinetic rate constants in the interval 0.1-10 fold the nominal value (Figure 2 bottom panel on the left), it can be seen that modulation in the efficiency of IRAK1 has a significant effect in IL-10 while it has a very small one in IL-6. In contract modulation of the IKK mediated has effect only on IL-6. The bottom panel on the right of Figure 2 expands the view of different combination of mutations on IKK and IRAK1. In this figure we can find all the conditions shown in the previous panels; four red crosses represent the single mutations displayed in panel top right, together with the wild type condition represented by the black cross. Red lines mark the single mutation values that are represented in the bottom left panel, and correspondingly indicated with the arrows. Finally, the white cross indicates a possible mutation that could explain the discrepancy shown in table 1 in the effect of IKK mutation on the production of IL-6, as through the white line the production of IL-6 does not depend on the value of the mutation of IRAK.

Sensitivity analysis

One of the key advantages of ODE models is the existence of a large array of computational and theoretical techniques to analyze the regulation of the system under investigation. An interesting one is sensitivity analysis, by which one can get quantitative information on how variation in the value of given model parameters or input signals can affect the dynamics and values of a given time-dependent variable(20). Here we focus on local sensitivities, which are calculated in a narrow region around the condition of interest. It is also possible to obtain a whole view of the sensitivity values for a width range of possible biological condition, in which case we refer to global sensitivities(21). This tool allows for detecting key model parameters and their linked biological processes.


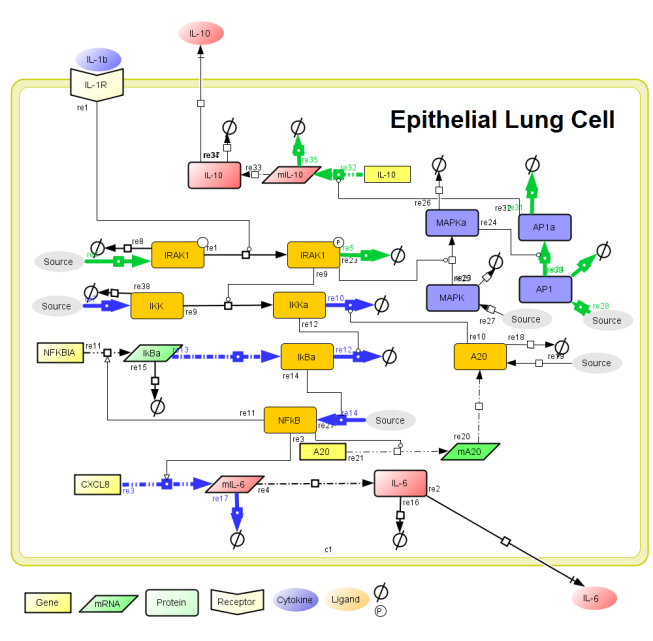


Figure 3. Sensitivities of the parameters for changes on the value of IL-6 and IL-10. Wide blue arrows indicate the most sensitivities parameters for changes on IL-6, wide green arrows show the parameters with higher sensitivity values for changes on IL-10.

Here we computed the local sensitivities (parameter values are varied within a small interval around its nominal value) for the maximal value of IL-6 and IL-10 as a measure of the production of these cytokines. In Figure 3 the results are displayed: the most sensitive parameters/processes are highlighted in thick blue and green arrows (for IL-6 and IL-10, respectively). As it can be seen in Figure 3, the production of IRAK is more sensitive to IL-10 variations, and production of IKK is more sensitive to IL-6 variations. These results converge with the simulations shown in Figure 2. IRAK1 intervenes in the production of IL-10 through MAPK pathway, but IKK does not which can explain the difference in sensitivities. The output of this analysis could be used to select promising molecular targets to increase or decrease the production of these interleukins.

**Case Study of Boolean modeling simulation: NFκB network activated in inflammatory macrophages.** Upon interaction of macrophages (MΦs) with pathogens, a large signaling and transcriptional regulatory network is activated. For the purpose of illustration, we selected the small part of the network proposed by Wentker(22) which accounts for the activation of NF-κB in response to Toll-like receptor 5 (TLR5) activation upon sensing bacterial flagellin protein. In the network, nodes represent miRNAs, proteins and other molecules, while edges represent their mutual interactions. Further, nodes representing cellular phenotypes triggered by the network are connected to their activator molecules and included in the extracellular space. The network has been created with CellDesigner(23) software like the original one from which it is extracted. The biological compounds can be distinguished by their shape: rectangles represent genes, rectangles with rounded corners proteins and complexes, parallelograms RNAs (both mRNAs and microRNAs), ellipses and circles small molecules, and hexagons phenotypes. In order to represent intracellular communications, CellDesigner allows for drawing cellular compartments: in Figure 4, two containers are depicted representing nuclear and outer membrane so to define cytoplasm, nucleus and extracellular compartment.


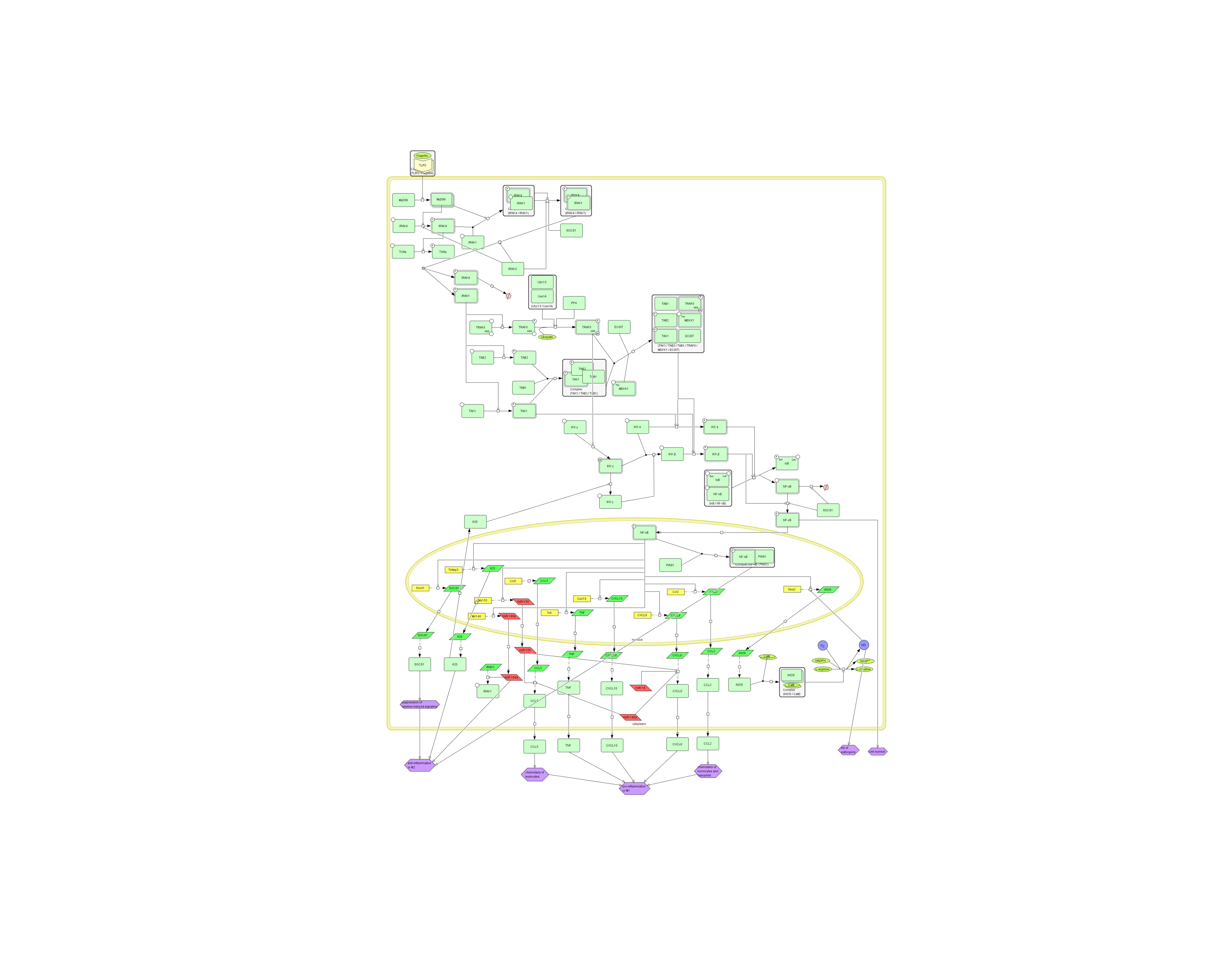


Figure 4: CellDesigner network of the NF-κB signaling pathway, transcriptional circuit and phenotypes layer. In this representation it is possible to clearly identify all cellular components by the color and the shape.

In the network, the biological trigger is represented by flagellin protein that binds to TLR5 and induces an intracellular signal cascade involving central signal transducing proteins like MyD88 or IRAK1. The signaling wired through these mediators converges in the activation of the IKKs which results in phosphorylation of IκB, the master inhibitor of NF-κB. This event is followed by IκB degradation and the release of NF-κB, which translocates to the nucleus and triggers the transcription of a large array of genes. Interestingly, among them there are negative NF-κB regulators like A20 and therefore the system constitute a well-studied case of negative feedback loop. The NF-κB -regulated genes include a broad range of pro-inflammatory cytokines and chemokines (TNF and CXCL10, respectively), but also negative regulators that promote the resolution of inflammation such as SOCS1 and A20 and miRNAs, some of them involved in negative feedback loops counterbalancing NF-κB activation ( miR-155, miR-146a)(24,25)**.** Further the regulatory network is connected to critical MΦ cellular phenotypes modulated via NF-κB signaling, including: 1) pro-inflammatory (differentiation into M1 subtype MΦ) and 2) anti-inflammatory (differentiation into M1 subtype of MΦ), 3) chemokine-mediated recruitment of immune cells, and 4) pathogen killing. In total, the network contains seven phenotypes, ten genes, three miRNAs, ions, simple molecules and several proteins. From the biological perspective, the network can be divided in three parts: input signals that trigger the signaling cascades (here the sensing of flagellin protein), an intermediate layer containing all the intracellular signaling (located in the cytoplasm) and transcriptional pathways (located in the nucleus), and an output layer with the cell phenotypes (located in the extracellular space).

To illustrate how the model simulation operates, we implemented a simulation in which flagellin protein bound TLR5 thus activating the downstream signaling, upstream cascade inhibitors IRAK3 and PP4 are knockdown, inhibitor SOCS1 is not expressed at the beginning of the simulation, and downstream NF-κB gene targets were transducible (Socs1, Tnfaip3, Mir155, Mir146, Ccl5, Tnf, Cxcl10, CXCL8, Ccl2, Nos2).

The simulation here presented has been computed in a Python script that first extracts the biological network from CellDesigner file, generates the equivalent Boolean model, and eventually evaluates the results for the given scenario. The Boolean simulation has been performed with Python module BooleanNet by Albert et al (26).

These and other simulations can be visualized as animated gif files at <http://sysbiomed-erlangen.weebly.com/resources.html>


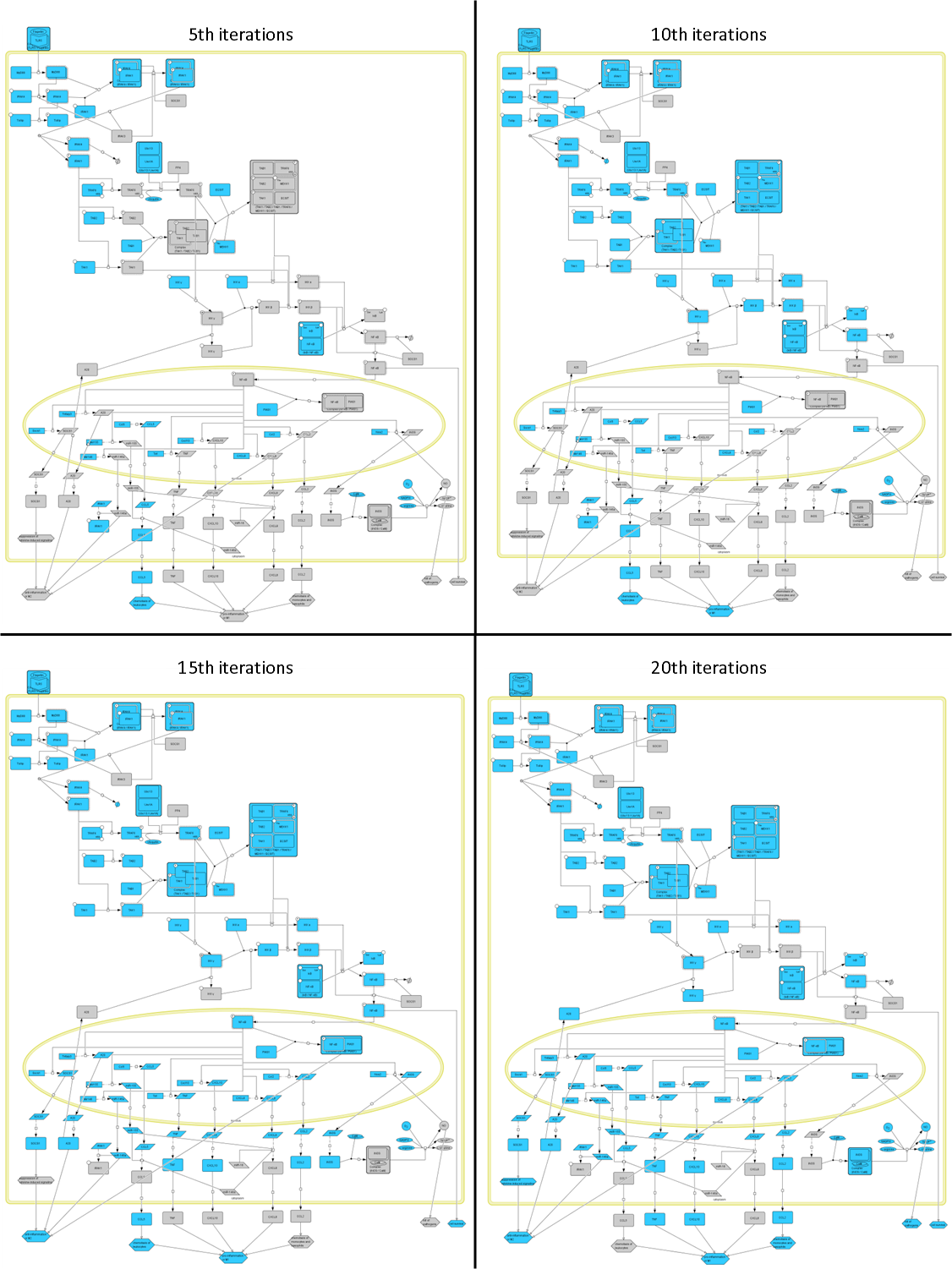


Figure 5: Propagation of the signal in the network during the simulation. Here we propose an overview of the state of the network at four different time point of the simulation. The blue color indicates nodes in an active state, while grey color represents non-active nodes. By starting at 5^th^ iteration (top-left corner), the signal has already propagated but it requires more than 10 iterations to reach Iκκs proteins and to activate NF-κB. In fact, at 10^th^ iteration (top-right corner) IKKγ has just been activated and has still to propagate and to activate IKKβ together with IKKα. In the following time point (bottom-left, 15^th^ iteration) we can see that the signal has propagated from NF-κ B within the nucleus and NF-κ B target genes have been translated and transcribed into the correspondent protein. To appreciate the effect of the transcribed proteins, especially A20 protein and the consequent inactivation of NF-κ B, more iterations are required (bottom-right, 20^th^ iteration).

Given these initial conditions, the signal propagates downstream updating the state of the nodes till the simulation reaches the maximum number of iterations (see Figure 1). In order to appreciate the presence of the oscillating state caused by the negative feedback loop and observe at least two complete cycles, we fixed the maximum number of iterations to 47. The model enters the oscillation state at the eighth iteration and requires eighteen more iterations to complete a full cycle (activation of IKKβ, translocation of NF-κB to the nucleus, transcription and translation of the target genes and consequent inactivation of NF-κB caused by A20 active protein followed by the inactivation of the downstream targets of NF-κB) and returning at the initial point of the oscillation. The model oscillation seems to reproduce the natural *in vivo* oscillation of the regulation of NF-κB through the negative feedback loop that we propose.

The oscillating state of the whole model includes the variation of the states of the phenotypes. In fact, the majority of the phenotypes follows an oscillating behavior determined by the direct dependence on the state of the downstream targets of NF-κB: "Chemotaxis of leukocytes", and subsequently the phenotype "Pro-inflammation" (directly triggered by the first) are activated and maintain the active state until upstream nodes (phenotypes “Chemotaxis of Leukocytes” and “Chemotaxis of Monocytes and Basophils” and proteins TNF, CXCL10 and CXCL8) turn to inactive state. From time iteration 15 on, “Pro-inflammation” remains active and the number of active proteins connected to this phenotype increased due to the propagation of the triggering signal. The phenotype “Pro-inflammation” turns inactive only if all of its upstream activators become inactive.

The complexity and computational requirements of Boolean models depend on the set of logic rules and on the number of elements included, and they usually are more restrained compared to other modeling approaches. For this reason, Boolean models can be used to predict in a systematic manner the response of the given network and to study qualitatively the effect of large number of gene knockouts(27). Therefore we have investigated the effect of systematic gene knockouts of the downstream targets of NF-κB by using the presented scenario as wild type reference situation, and perturbed the model by knocking down pair of genes. We run a simulation for each couple of knocked down genes and set the maximum iteration number to 27 so to cover one entire oscillating cycle. We again assumed that the flagellin protein was continuously binding to TLR5 for all 27 iterations by ensuring a continuous stimulus. The aim of the simulation was first identifying variations in the activation state of chemokines Ccl5, Ccl2, Cxcl10, Cxcl8 and the seven phenotypes, and eventually clustering the network response. Since Boolean modeling is more qualitative than quantitative, for measuring the difference between the wild type condition and the knockout simulation we counted the number of consecutive iterations in which each element of interest was in an active state. We then computed the difference between the reference condition and each knockout independently.

In Figure 6 we present the final results of the systematic knockouts of pair of genes. Each row represents a knockout of a pair of genes, and each column a phenotype or a chemokine of interest. Blue color indicates that the number of consecutive active iteration for the considered phenotype or chemokine is decreased in the knockout scenario compared to the reference wild type one. Red color indicates an increased number of consecutive active states. It is interesting to observe that for the knockout of some pairs of genes the network shows the same response. As example, we highlighted this behavior for three genes: miR-155, TNFAIP3 and CCL2 genes. On the right side of the heatmap, we indicated the rows that represent the knockout of the considered gene with a colored square. The unsupervised hierarchical clustering identified common behavior for some groups of knockout simulations and created a cluster, which we indicated with a continuous colored bar on the right side for the three candidate genes. From the heatmap we can infer that some genes are more capable of influencing the activation state of phenotypes and chemokines, and the cluster identified can be liked back to the knockout of one specific gene of the pair. When both genes are equally strong, we observe an intermediate behavior that presents part of the features of one gene knockout and part of the features of the other one. For example the knockout combination of miR-155 gene with TNFAIP3 gene upregulates all chemokines activation and five on seven phenotypes, thus summing the effect of the single gene knockout. By combining Tnfaip3 knockout with Ccl2 we see that “Chemotaxis monocytes basophils” phenotype and Ccl2 secretion are downregulated, behavior that is associated to Ccl2 knockout more than to Tnfaip3 knockout. In this case we do not see the sum of effects but rather a gene knockout that prevails on the other. This effect is strongly determined by the wiring that connects the elements of the model.


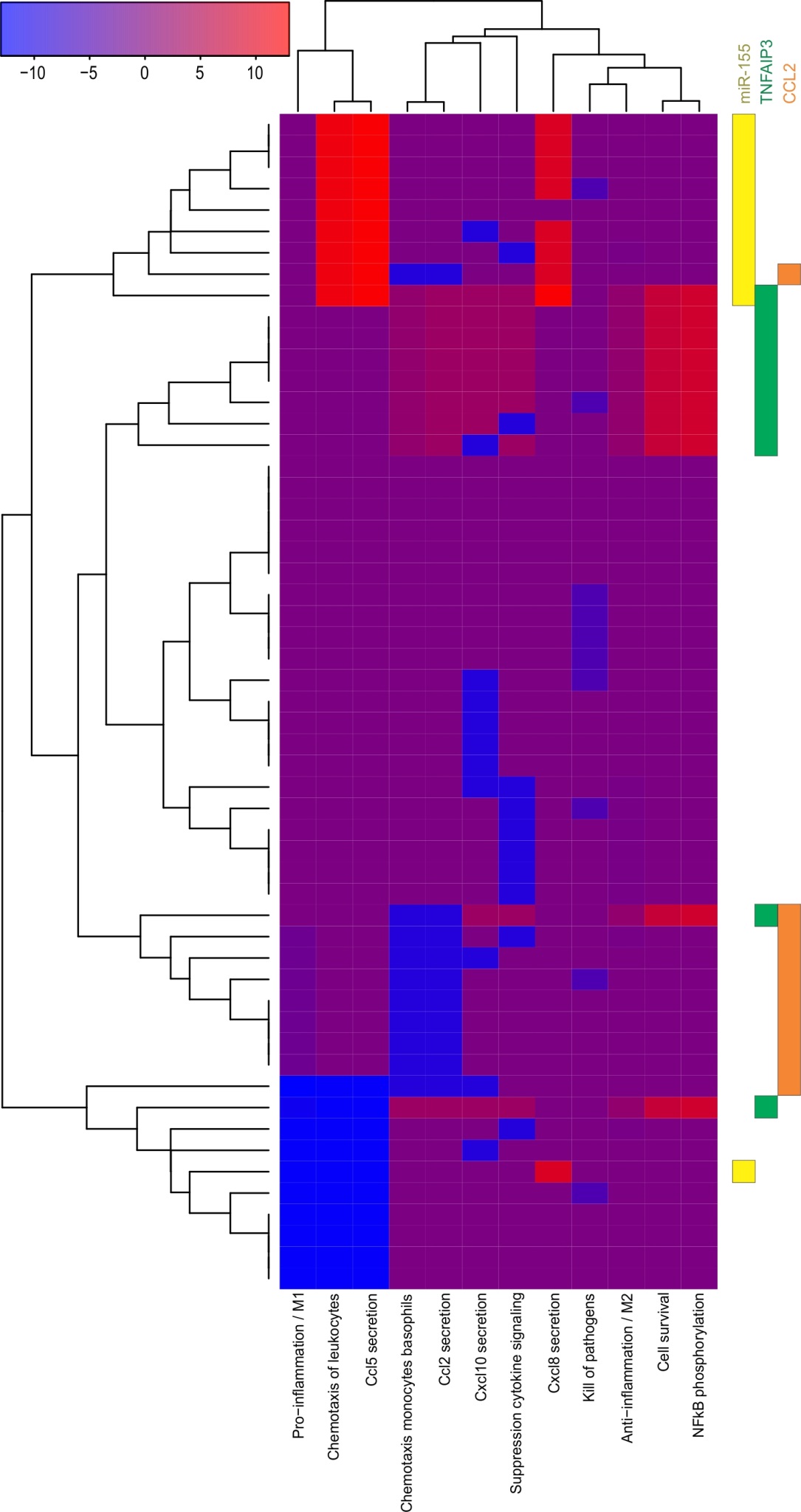


Figure 6: Heatmap representing the difference of the activation state of phenotypes for different initial conditions set. Unsupervised hierarchical clustering on rows shows that specific knockout combinations forms clusters that can be associated to a biological behavior. The presence of clusters suggests that some genes are more capable of influencing the outcome of the simulation: for example, we identified three clusters where the activation state of the phenotypes can be easily led back to the knockout of a specific gene even if the simulation included two distinct knockouts. Yellow squares at right side of the heatmap represent the cluster where knockout of miR-155 gene is the prominent knockout, green squares are associated to TNFAIP3 gene knockout, while orange squares are associated to Ccl2 gene knockout.

1. Resasco DC, Gao F, Morgan F, Novak IL, Schaff JC, Slepchenko BM. Virtual Cell: computational tools for modeling in cell biology. Wiley Interdiscip Rev Syst Biol Med. 2012 Apr;4(2):129–40.

2. Hao W, Schlesinger LS, Friedman A. Modeling Granulomas in Response to Infection in the Lung. PloS One. 2016;11(3):e0148738.

3. Fisher J, Henzinger TA. Executable cell biology. Nat Biotechnol. 2007 Nov;25(11):1239–49.

4. Rohr C, Marwan W, Heiner M. Snoopy--a unifying Petri net framework to investigate biomolecular networks. Bioinforma Oxf Engl. 2010 Apr 1;26(7):974–5.

5. Efroni S, Harel D, Cohen IR. Emergent Dynamics of Thymocyte Development and Lineage Determination. PLoS Comput Biol [Internet]. 2007 Jan [cited 2017 Mar 3];3(1). Available from: http://www.ncbi.nlm.nih.gov/pmc/articles/PMC1782042/

6. Peng SC, Wong DSH, Tung KC, Chen YY, Chao CC, Peng CH, et al. Computational modeling with forward and reverse engineering links signaling network and genomic regulatory responses: NF-kappaB signaling-induced gene expression responses in inflammation. BMC Bioinformatics. 2010 Jun 8;11:308.

7. Carvalho RV, van den Heuvel J, Kleijn J, Verbeek FJ. Coupling of Petri Net Models of the Mycobacterial Infection Process and Innate Immune Response. Computation. 2015 Apr 8;3(2):150–76.

8. Baart GJE, Martens DE. Genome-scale metabolic models: reconstruction and analysis. Methods Mol Biol Clifton NJ. 2012;799:107–26.

9. Bordbar A, Monk JM, King ZA, Palsson BO. Constraint-based models predict metabolic and associated cellular functions. Nat Rev Genet. 2014 Feb;15(2):107–20.

10. Ferrarini MG, Siqueira FM, Mucha SG, Palama TL, Jobard É, Elena-Herrmann B, et al. Insights on the virulence of swine respiratory tract mycoplasmas through genome-scale metabolic modeling. BMC Genomics. 2016 May 13;17:353.

11. Hoffmann A, Levchenko A, Scott ML, Baltimore D. The IkappaB-NF-kappaB signaling module: temporal control and selective gene activation. Science. 2002 Nov 8;298(5596):1241–5.

12. Krishna S, Jensen MH, Sneppen K. Minimal model of spiky oscillations in NF-kappaB signaling. Proc Natl Acad Sci U S A. 2006 Jul 18;103(29):10840–5.

13. Lipniacki T, Paszek P, Brasier ARAR, Luxon B, Kimmel M. Mathematical model of NF-kappaB regulatory module. J Theor Biol. 2004 May 21;228(2):195–215.

14. Ashall L, Horton CA, Nelson DE, Paszek P, Harper CV, Sillitoe K, et al. Pulsatile stimulation determines timing and specificity of NF-kappaB-dependent transcription. Science. 2009 Apr 10;324(5924):242–6.

15. Johnson GL, Lapadat R. Mitogen-activated protein kinase pathways mediated by ERK, JNK, and p38 protein kinases. Science. 2002 Dec 6;298(5600):1911–2.

16. Davidson DJ, Currie AJ, Bowdish DME, Brown KL, Rosenberger CM, Ma RC, et al. IRAK-4 mutation (Q293X): rapid detection and characterization of defective post-transcriptional TLR/IL-1R responses in human myeloid and non-myeloid cells. J Immunol Baltim Md 1950. 2006 Dec 1;177(11):8202–11.

17. Koziczak-Holbro M, Joyce C, Glück A, Kinzel B, Müller M, Tschopp C, et al. IRAK-4 kinase activity is required for interleukin-1 (IL-1) receptor- and toll-like receptor 7-mediated signaling and gene expression. J Biol Chem. 2007 May 4;282(18):13552–60.

18. Filipe-Santos O, Bustamante J, Haverkamp MH, Vinolo E, Ku C-L, Puel A, et al. X-linked susceptibility to mycobacteria is caused by mutations in NEMO impairing CD40-dependent IL-12 production. J Exp Med. 2006 Jul 10;203(7):1745–59.

19. Bustamante J, Boisson-Dupuis S, Jouanguy E, Picard C, Puel A, Abel L, et al. Novel primary immunodeficiencies revealed by the investigation of paediatric infectious diseases. Curr Opin Immunol. 2008 Feb;20(1):39–48.

20. Saltelli A, Tarantola S, Campolongo F. Sensitivity Analysis as an Ingredient of Modeling. Stat Sci. 2000;15(4):377–95.

21. Mathew S, Bartels J, Banerjee I, Vodovotz Y. Global sensitivity analysis of a mathematical model of acute inflammation identifies nonlinear dependence of cumulative tissue damage on host interleukin-6 responses. J Theor Biol. 2014 Oct 7;358:132–48.

22. Wentker P, Eberhardt M, Dreyer FS, Bertrams W, Cantone M, Griss K, et al. An Interactive Macrophage Signal Transduction Map Facilitates Comparative Analyses of High-Throughput Data. J Immunol Baltim Md 1950. 2017 Mar 1;198(5):2191–201.

23. Akira Funahashi MM. CellDesigner: A process diagram editor for gene-regulatory and biochemical networks. Biosilico. 2003;1(5).

24. Hou J, Wang P, Lin L, Liu X, Ma F, An H, et al. MicroRNA-146a feedback inhibits RIG-I-dependent Type I IFN production in macrophages by targeting TRAF6, IRAK1, and IRAK2. J Immunol Baltim Md 1950. 2009 Aug 1;183(3):2150–8.

25. Lindsay MA. microRNAs and the immune response. Trends Immunol. 2008 Jul;29(7):343–51.

26. Albert I, Thakar J, Li S, Zhang R, Albert R. Boolean network simulations for life scientists. Source Code Biol Med. 2008;3:16.

27. Saez-Rodriguez J, Simeoni L, Lindquist JA, Hemenway R, Bommhardt U, Arndt B, et al. A logical model provides insights into T cell receptor signaling. PLoS Comput Biol. 2007 Aug;3(8):e163.

# REFERENCES

1. Resasco DC, Gao F, Morgan F, Novak IL, Schaff JC, Slepchenko BM. Virtual Cell: computational tools for modeling in cell biology. Wiley Interdiscip Rev Syst Biol Med. 2012 Apr;4(2):129–40.

2. Hao W, Schlesinger LS, Friedman A. Modeling Granulomas in Response to Infection in the Lung. PloS One. 2016;11(3):e0148738.

3. Fisher J, Henzinger TA. Executable cell biology. Nat Biotechnol. 2007 Nov;25(11):1239–49.

4. Rohr C, Marwan W, Heiner M. Snoopy--a unifying Petri net framework to investigate biomolecular networks. Bioinforma Oxf Engl. 2010 Apr 1;26(7):974–5.

5. Efroni S, Harel D, Cohen IR. Emergent Dynamics of Thymocyte Development and Lineage Determination. PLoS Comput Biol [Internet]. 2007 Jan [cited 2017 Mar 3];3(1). Available from: http://www.ncbi.nlm.nih.gov/pmc/articles/PMC1782042/

6. Peng SC, Wong DSH, Tung KC, Chen YY, Chao CC, Peng CH, et al. Computational modeling with forward and reverse engineering links signaling network and genomic regulatory responses: NF-kappaB signaling-induced gene expression responses in inflammation. BMC Bioinformatics. 2010 Jun 8;11:308.

7. Carvalho RV, van den Heuvel J, Kleijn J, Verbeek FJ. Coupling of Petri Net Models of the Mycobacterial Infection Process and Innate Immune Response. Computation. 2015 Apr 8;3(2):150–76.

8. Baart GJE, Martens DE. Genome-scale metabolic models: reconstruction and analysis. Methods Mol Biol Clifton NJ. 2012;799:107–26.

9. Bordbar A, Monk JM, King ZA, Palsson BO. Constraint-based models predict metabolic and associated cellular functions. Nat Rev Genet. 2014 Feb;15(2):107–20.

10. Ferrarini MG, Siqueira FM, Mucha SG, Palama TL, Jobard É, Elena-Herrmann B, et al. Insights on the virulence of swine respiratory tract mycoplasmas through genome-scale metabolic modeling. BMC Genomics. 2016 May 13;17:353.

11. Hoffmann A, Levchenko A, Scott ML, Baltimore D. The IkappaB-NF-kappaB signaling module: temporal control and selective gene activation. Science. 2002 Nov 8;298(5596):1241–5.

12. Krishna S, Jensen MH, Sneppen K. Minimal model of spiky oscillations in NF-kappaB signaling. Proc Natl Acad Sci U S A. 2006 Jul 18;103(29):10840–5.

13. Lipniacki T, Paszek P, Brasier ARAR, Luxon B, Kimmel M. Mathematical model of NF-kappaB regulatory module. J Theor Biol. 2004 May 21;228(2):195–215.

14. Ashall L, Horton CA, Nelson DE, Paszek P, Harper CV, Sillitoe K, et al. Pulsatile stimulation determines timing and specificity of NF-kappaB-dependent transcription. Science. 2009 Apr 10;324(5924):242–6.

15. Johnson GL, Lapadat R. Mitogen-activated protein kinase pathways mediated by ERK, JNK, and p38 protein kinases. Science. 2002 Dec 6;298(5600):1911–2.

16. Davidson DJ, Currie AJ, Bowdish DME, Brown KL, Rosenberger CM, Ma RC, et al. IRAK-4 mutation (Q293X): rapid detection and characterization of defective post-transcriptional TLR/IL-1R responses in human myeloid and non-myeloid cells. J Immunol Baltim Md 1950. 2006 Dec 1;177(11):8202–11.

17. Koziczak-Holbro M, Joyce C, Glück A, Kinzel B, Müller M, Tschopp C, et al. IRAK-4 kinase activity is required for interleukin-1 (IL-1) receptor- and toll-like receptor 7-mediated signaling and gene expression. J Biol Chem. 2007 May 4;282(18):13552–60.

18. Filipe-Santos O, Bustamante J, Haverkamp MH, Vinolo E, Ku C-L, Puel A, et al. X-linked susceptibility to mycobacteria is caused by mutations in NEMO impairing CD40-dependent IL-12 production. J Exp Med. 2006 Jul 10;203(7):1745–59.

19. Bustamante J, Boisson-Dupuis S, Jouanguy E, Picard C, Puel A, Abel L, et al. Novel primary immunodeficiencies revealed by the investigation of paediatric infectious diseases. Curr Opin Immunol. 2008 Feb;20(1):39–48.

20. Saltelli A, Tarantola S, Campolongo F. Sensitivity Analysis as an Ingredient of Modeling. Stat Sci. 2000;15(4):377–95.

21. Mathew S, Bartels J, Banerjee I, Vodovotz Y. Global sensitivity analysis of a mathematical model of acute inflammation identifies nonlinear dependence of cumulative tissue damage on host interleukin-6 responses. J Theor Biol. 2014 Oct 7;358:132–48.

22. Wentker P, Eberhardt M, Dreyer FS, Bertrams W, Cantone M, Griss K, et al. An Interactive Macrophage Signal Transduction Map Facilitates Comparative Analyses of High-Throughput Data. J Immunol Baltim Md 1950. 2017 Mar 1;198(5):2191–201.

23. Akira Funahashi MM. CellDesigner: A process diagram editor for gene-regulatory and biochemical networks. Biosilico. 2003;1(5).

24. Hou J, Wang P, Lin L, Liu X, Ma F, An H, et al. MicroRNA-146a feedback inhibits RIG-I-dependent Type I IFN production in macrophages by targeting TRAF6, IRAK1, and IRAK2. J Immunol Baltim Md 1950. 2009 Aug 1;183(3):2150–8.

25. Lindsay MA. microRNAs and the immune response. Trends Immunol. 2008 Jul;29(7):343–51.

26. Albert I, Thakar J, Li S, Zhang R, Albert R. Boolean network simulations for life scientists. Source Code Biol Med. 2008;3:16.

27. Saez-Rodriguez J, Simeoni L, Lindquist JA, Hemenway R, Bommhardt U, Arndt B, et al. A logical model provides insights into T cell receptor signaling. PLoS Comput Biol. 2007 Aug;3(8):e163.
